# Inter- and intratumor DNA methylation heterogeneity associated with lymph node metastasis and prognosis of esophageal squamous cell carcinoma

**DOI:** 10.7150/thno.42559

**Published:** 2020-02-10

**Authors:** Huajing Teng, Meiying Xue, Jialong Liang, Xingxing Wang, Lu Wang, Wenqing Wei, Chao Li, Ze Zhang, Qinglan Li, Xia Ran, Xiaohui Shi, Wanshi Cai, Weihu Wang, Hengjun Gao, Zhongsheng Sun

**Affiliations:** 1 Institute of Genomic Medicine, Wenzhou Medical University, Wenzhou, China; 2 Beijing Institutes of Life Science, Chinese Academy of Sciences, Beijing, China; 3 Key Laboratory of Carcinogenesis and Translational Research (Ministry of Education/Beijing), Department of Radiation Oncology, Peking University Cancer Hospital & Institute, Beijing, China; 4 University of Chinese Academy of Sciences, Beijing, China; 5 Institute of Digestive Diseases, Tongji University School of Medicine, Shanghai, China; 6 National Engineering Center for Biochip at Shanghai, Shanghai, China

**Keywords:** epigenetic heterogeneity, cancer mortality, whole-genome bisulfite sequencing, prognostic markers, personalized medicine

## Abstract

**Background**: Esophageal squamous cell carcinoma (ESCC), one of the leading causes of cancer mortality worldwide, is a heterogeneous cancer with diverse clinical manifestations. However, little is known about the epigenetic heterogeneity and its clinical relevance for this prevalent cancer.

**Methods**: We generated 7.56 Tb single-base resolution whole-genome bisulfite sequencing data for 84 ESCC and paired paraneoplastic tissues. The analysis identified inter- and intratumor DNA methylation (DNAm) heterogeneity, epigenome-wide DNAm alterations together with the functional regulators involved in the hyper- or hypomethylated regions, and their association with clinical features. We then validated the correlation between the methylation level of specific regions and clinical outcomes of 96 ESCC patients in an independent cohort.

**Results**: ESCC manifested substantial inter- and intratumor DNAm heterogeneity. The high intratumor DNAm heterogeneity was associated with lymph node metastasis and worse overall survival. Interestingly, hypermethylated regions in ESCC were enriched in promoters of numerous transcription factors, and demethylated noncoding regions related to RXR transcription factor binding appeared to contribute to the development of ESCC. Furthermore, we identified numerous DNAm alterations associated with carcinogenesis and lymph node metastasis of ESCC. We also validated three novel prognostic markers for ESCC, including one each in the promoter of *CLK1*, the 3' untranslated region of *ZEB2,* and the intergenic locus surrounded by several lncRNAs.

**Conclusions**: This study presents the first population-level resource for dissecting base-resolution DNAm variation in ESCC and provides novel insights into the ESCC pathogenesis and progression, which might facilitate diagnosis and prognosis for this prevalent malignancy.

## Introduction

Esophageal cancer is the ninth most prevalent malignancy and the sixth leading cause of cancer mortality worldwide 1. Esophageal squamous cell carcinoma (ESCC), which constitutes greater than 80% of esophageal cancers, represents the most frequent histological type of esophageal cancer 2, and the incidence and mortality of ESCC exhibit considerable geographic variation 3, 4. The majority of ESCC patients experience lymph node metastasis (LNM) or distal metastases at diagnosis, leading to poor outcomes for these patients (five-year survival rate < 20%) 2, 5. Large-scale exome and genome sequencing have identified numerous genomic alterations in ESCC, including somatic mutations in *TP53*, *PIK3CA*, *NOTCH1*, and copy number alterations (CNAs) in pivotal RTK-MAPK-PI3K pathway genes 4, 6-10. This information has yielded profound insights for precision diagnosis and treatment of this common cancer. However, genetic alterations alone are inadequate in explaining the complexity, prevalence, and pathogenesis of ESCC.

ESCC has been reported to harbor abundant inactivating mutations in histone-modifying and chromatin-remodeling regulators, KMT2D (MLL2), KDM6A (UTX), and KMT2C (MLL3) 11. Genetic mutations in these epigenetic regulators might disrupt the entire epigenetic regulatory network, highlighting the significance of adjusting our focus beyond genetic alterations in ESCC pathogenesis. DNA methylation (DNAm), one of the well-characterized epigenetic modifications, coordinates various biological processes by regulating gene expression and posttranscriptional activity 12-14. Some specific DNAm alterations have been established as hallmarks in specific cancers and can be used in the diagnosis of specific cancer types 15-20. In particular, abnormal DNAm in oncogenes and tumor-suppressor genes are generally involved in all steps of tumorigenesis 21-23. Compared to traditional irreversible genetic changes, DNAm can be reversed with drugs 24, 25, indicating their potential role as molecular targets for therapeutic intervention. Previous studies have investigated DNAm changes in ESCC based on methylation array data from The Cancer Genome Atlas (TCGA) project, identifying several specific DNAm alterations associated with clinical outcomes in ESCC patients 26, 27. However, array-based technologies limit exhaustive screening of epigenome-wide DNAm alterations due to low genome coverage and low sensitivity of probe cross-hybridization. Thus, it is imperative to decipher the epigenome-wide high-resolution DNAm signatures of ESCC for clinical applications.

Inter- and intratumor genetic heterogeneity is a fundamental property of human cancers that confers a formidable barrier to cancer treatment 28. Spatial genetic heterogeneity and clonal evolution in esophageal cancer have been demonstrated 29-31; however, inter- and intratumoral epigenetic heterogeneity and their clinical relevance for ESCC is less well defined. Recent data in other cancer types have shown the power of DNAm sequencing for analyzing epigenetic heterogeneity. DNAm heterogeneity, quantified using the proportion of discordantly methylated reads (PDR), entropy, or epipolymorphism, has been linked to clinical variables in acute myeloid leukemia 32, chronic lymphocytic leukemia 33, glioblastoma 34, and Ewing sarcoma 35, suggesting the extensive involvement of DNAm heterogeneity in tumorigenesis and progression.

In this study, we performed whole-genome bisulfite sequencing (WGBS) on 84 ESCCs and paired paraneoplastic tissues to elucidate epigenetic heterogeneity at both inter- and intratumor levels as well as their association with clinical outcomes. Furthermore, we identified epigenome-wide DNAm alterations associated with carcinogenesis and LNM of ESCC. Our study provides novel insights into the ESCC pathogenesis and progression, which might facilitate its diagnosis and prognosis.

## Materials and Methods

### Clinical patients and samples

Eighty-four ESCC and paired paraneoplastic samples were provided by the National Engineering Center for Biochip at Shanghai (Shanghai, China), whose pathological and clinical features are shown in **Supplementary Table S1**. All tumor samples were examined by two experienced pathologists and ensured the carcinoma content greater than 70%. Samples were snap-frozen in liquid nitrogen and stored at -80°C for WGBS and targeted DNA sequencing. The study was approved by the Ethical Review Board of National Engineering Center for Biochip at Shanghai (ID: YB M-05-02), and clinical data were collected after patients provided informed consent.

Clinical data of 96 ESCC patients and their matched methylation array data were downloaded from the TCGA-ESCA cohort (https://portal.gdc.cancer.gov).

### DNA extraction, targeted DNA sequencing, and identification of somatic mutations

Genomic DNA was extracted from frozen tissues using the DNeasy Blood & Tissue Kit (Qiagen, Shanghai, China) and was then used for targeted DNA sequencing and WGBS library construction. A mutation hotspot panel was designed to target 32 ESCC-related genes (**Supplementary Table S2**), which were reported to be frequently mutated in two cohorts 9, 11 or Cosmic database. Approximately 1 μg of genomic DNA was sheared using a Covaris S220 focused-ultrasonicator, resulting in fragments of 150~250 bp. End repair, dA-tailing, and adapter ligation were performed using KAPA Hyper Prep Kits. The ligation product was cleaned up and size-selected using Beckman Ampure XP Beads (Beckman Coulter, Brea, CA), followed by PCR to generate the whole-genome library, which was then hybridized with biotin-labeled probes of target regions and captured using Dynabeads™ MyOne™ Streptavidin T1, and amplified to generate targeted DNA library. Subsequently, the library was sequenced for Illumina 150-bp paired-end reads, and clean reads were aligned to a reference genome (hg38, GRCh38) using the Burrows-Wheeler Aligner (BWA) (http://bio-bwa.sourceforge.net/). After local realignment, somatic single nucleotide variants were identified using MuTect (https://software.broadinstitute.org/cancer/cga/mutect), and annotated using VarCards 36.

### WGBS and methylation analysis

MethylC-seq protocol was used to prepare WGBS sequencing libraries. First, approximately 1 μg genomic DNA was fragmented into 400 bp fragments using a Covaris S220 focused-ultrasonicator. Fragmented DNA was end-repaired, and dA tailed and then ligated to Illumina TruSeq adapter (all Cs methylated) using the KAPA Hyper Prep Kit (KAPA Biosystems, Wilmington, MA, USA). The adapter-ligated DNA was purified with beads and eluted with elution buffer. The EpiTect Fast DNA Bisulfite Kit (Qiagen, Shanghai, China) was used to convert the adapter-ligated DNA. Finally, bisulfite-treated DNA was amplified using 2× KAPA HiFi Uracil+ Readymix (KAPA Biosystems, Wilmington, MA, USA) to produce the WGBS library. Each library was sequenced using the Illumina HiSeq X Ten platform and generated 150 bp paired-end reads. Raw sequencing datasets were deposited in the Sequence Read Archive of NCBI (http://www.ncbi.nlm.nih.gov/sra) under accession number PRJNA523898. Sequencing quality was assessed using the FastQC software (Babraham Bioinformatics, Cambridge, UK). All sequencing reads were aligned to the human reference genome GRCh38/hg38 using BsMap (http://code.google.com/p/bsmap/). Methylation site calling and methylation levels of each CpG site were determined using BisSNPV.0.82.2 software (https://sourceforge.net/projects/bissnp). Whole-genome differentially methylated regions (DMRs) were detected using metilene software 37. The dimension reduction analysis of tumor and normal tissues based on the methylation levels of 53,995 reference genes (Ensembl v78) was performed using multidimensional scaling (MDS) in Euclidean distance with the R package RnBeads 38.

### Chromatin immunoprecipitation sequencing data analysis

ChIP-seq data of two ESCC cell lines (TE7, KYSE510) from a previous study 39 were mapped to the human reference genome (GRCh38/hg38) using Bowtie (http://bowtie-bio.sourceforge.net/index.shtml). ChIP-Seq peaks were determined with read pileups for every 50 bp bins using MACS (http://liulab.dfci.harvard.edu/MACS/). The generated wiggle files were normalized in terms of reads per million (rpm) and then transformed into bigwig format files using the wigToBigWig tool (http://hgdownload.cse.ucsc.edu/admin/exe/). H3K27ac bigwig tracks were visualized in the UCSC Genome Browser (http://genome.ucsc.edu/cgi-bin/hgTracks).

### Bioinformatic analysis of heterogeneity among tumor samples

Inter-individual DNAm heterogeneity quantified by the coefficient of variation (CV) of the tumor and paraneoplastic tissues was measured according to the previously described method 35. The methylation level of each 5-kb tiling region was averaged only when the region was covered by more than ten sequencing reads, and then the CV for each sample was calculated according to the methylation levels of these genome-wide tiling regions.

### Bioinformatic analysis of heterogeneity within individual tumors

Intratumor DNAm heterogeneity was quantified by PDR, entropy, or epipolymorphism. The PDR score was calculated as the proportion of discordant reads, containing both methylated and unmethylated CpGs, among all WGBS reads within a local region that covered at least four CpGs 33. PDR was determined only when each CpG was covered by more than ten reads. The epi-allele entropy and epipolymorphism were calculated using a slightly modified version of methclone (https://code.google.com/p/methclone). Input files to methclone were created by aligning the WGBS reads to the human reference genome (hg38) using Bismark (https://www.bioinformatics.babraham.ac.uk/projects/bismark/).

### Region set and functional enrichment analysis

Region set enrichment analysis among differentially methylated regions was determined using Locus Overlap Analysis (LOLA) 40. P-values were corrected for multiple testing using the Benjamini and Yekutieli method, and all enrichments with an adjusted p-value below 0.05 were considered significant. Functional enrichment analysis for host genes of promoter-associated DMRs was performed using BiNGO (https://www.psb.ugent.be/cbd/papers/BiNGO/Home.html) with an adjusted p-value below 0.05.

### Patient survival analysis

Kaplan-Meier survival was performed using the R package 'survival'. Significance in overall or disease-free survival was calculated using the log-rank test. Cox proportional hazards regression was performed using the function coxph () from the R package 'survival'.

### Somatic copy-number alteration analysis

Somatic CNAs of each paired tumor and paraneoplastic samples was performed using R package SaasCNV (https://zhangz05.u.hpc.mssm.edu/saasCNV/). Recurrent focal somatic CNAs were identified by the GISTIC 2.0 algorithm (http://software.broadinstitute.org/cancer/software/genepattern/modules/docs/GISTIC_2.0).

## Results

### Epigenome-wide alterations of DNAm in ESCC

**Figure 1:**
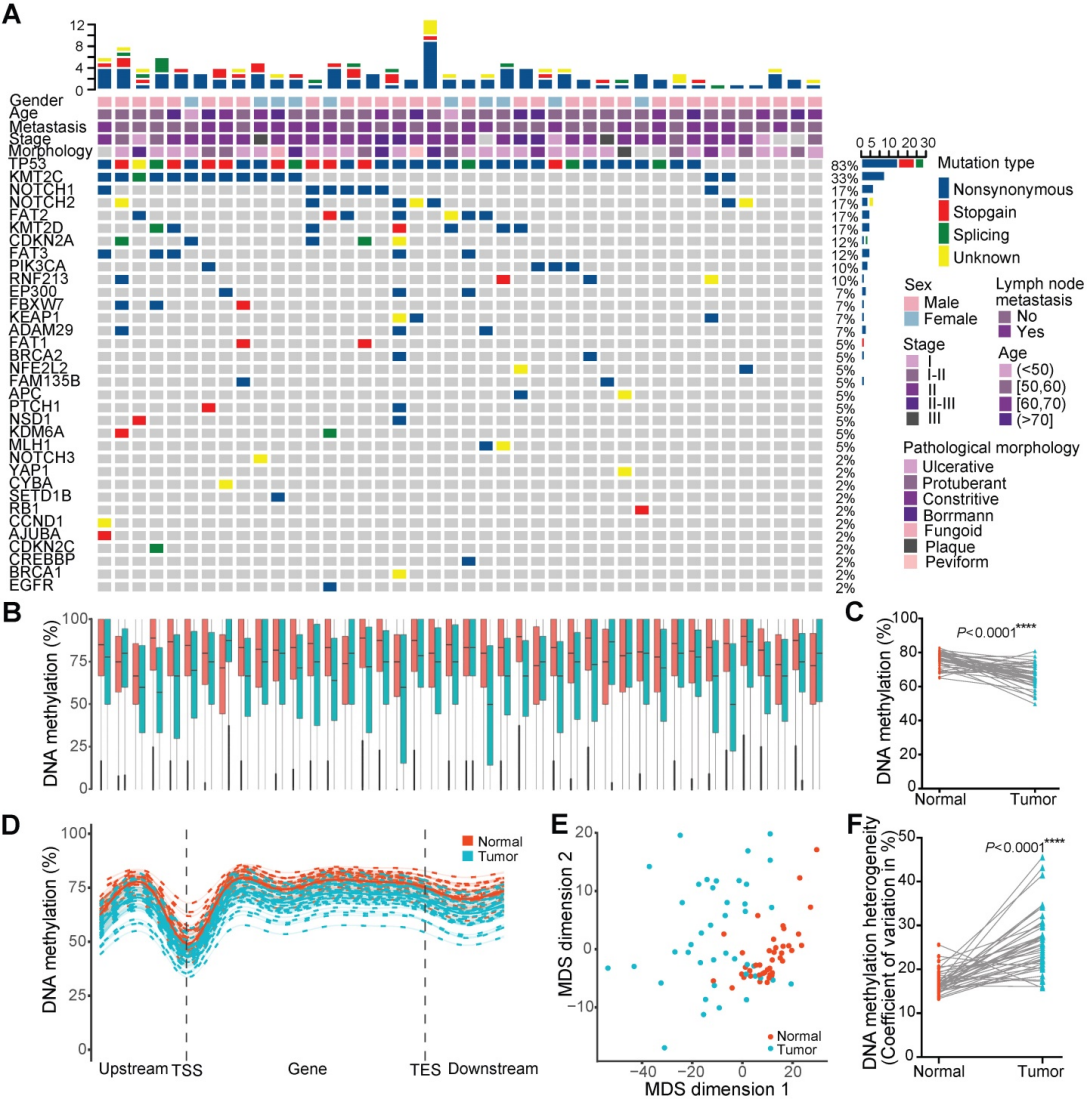
Epigenome-wide changes of DNA methylation in ESCC. (A) Landscape of somatic genetic mutations in 42 ESCC patients. Each row denotes a gene, and each column represents an individual tumor. The uppermost scale indicates the number of identified mutations (y-axis) for each patient (x-axis). The top five rows of the x-axis indicate key clinical parameters for each patient. The right side of the y-axis shows the percentage of samples with mutations for each gene. Mutation type and clinical characteristics are represented by different colors. DNA methylation (B) and global DNA methylation (C) levels between 42 tumors and paired normal tissues. (D) Metaplot of CpG methylation levels across gene bodies. TSS, transcription start site; TES, transcription end site. (E) Multidimensional scaling plot of the tumor and normal tissues based on the methylation levels of all reference genes. (F) Interindividual DNA methylation heterogeneity quantified by the coefficient of variation between 42 tumors and paired normal tissues.

Single-base resolution DNAm profiles can provide an unbiased global view of the DNAm landscape. To gain a comprehensive insight into variations in the DNA methylome in ESCC, we performed WGBS on 84 ESCCs and paired paraneoplastic tissues from 42 patients with a median age of 58 and median overall survival of 9.18 months (**Figure 1A**). Seventy-one percent of these patients were TNM stage II, and 48% had LNM. Targeted DNA amplicon sequencing showed 83% of them harbored TP53 mutations (**Figure 1A**). Over 7.56 Tb sequencing data produced by WGBS from these patients were mapped to a reference genome (Hg38) using Bsmap, providing a median coverage of 24.92× per sample (range 17.04-39.77×; **Supplementary Table 1**). Epigenome-wide alterations of DNAm were observed in ESCC tissues, and the average CpG methylation levels of normal paraneoplastic tissues and cancer tissues were 76.31% and 66.36%, respectively (**Figure 1B, C**). In general, the methylation levels of transcription start sites were the lowest, but with the marked elevation in the gene body region in both tissues. The global methylation levels in all regions were systematically reduced in cancer tissues compared to paraneoplastic tissues (**Figure 1D**). Multidimensional scaling analysis based on the methylation level of all reference genes was performed to assess the similarity of individuals within the dataset. The multidimensional scaling plot discriminated the majority of malignant and adjacent benign tissues from ESCC patients, with normal tissues clustering closer together than cancer tissues (**Figure 1E**), indicating highly variable methylation patterns across cancer tissues. These findings were confirmed by interindividual heterogeneity quantified using the coefficient of variation for DNAm level throughout the genome (**Figure 1F**). The average DNAm heterogeneity level in ESCC patients was 0.26, with a range from 0.16 to 0.46, which is higher than that of several recognized heterogeneous cancer types, including prostate cancer and chronic lymphocytic leukemia (CLL) 35. These results revealed substantial DNAm heterogeneity among ESCC patients, highlighting the importance of considering nongenetic aspects of tumor heterogeneity in pathogenesis and therapy of ESCC.

### DNAm alterations associated with ESCC carcinogenesis

**Figure 2:**
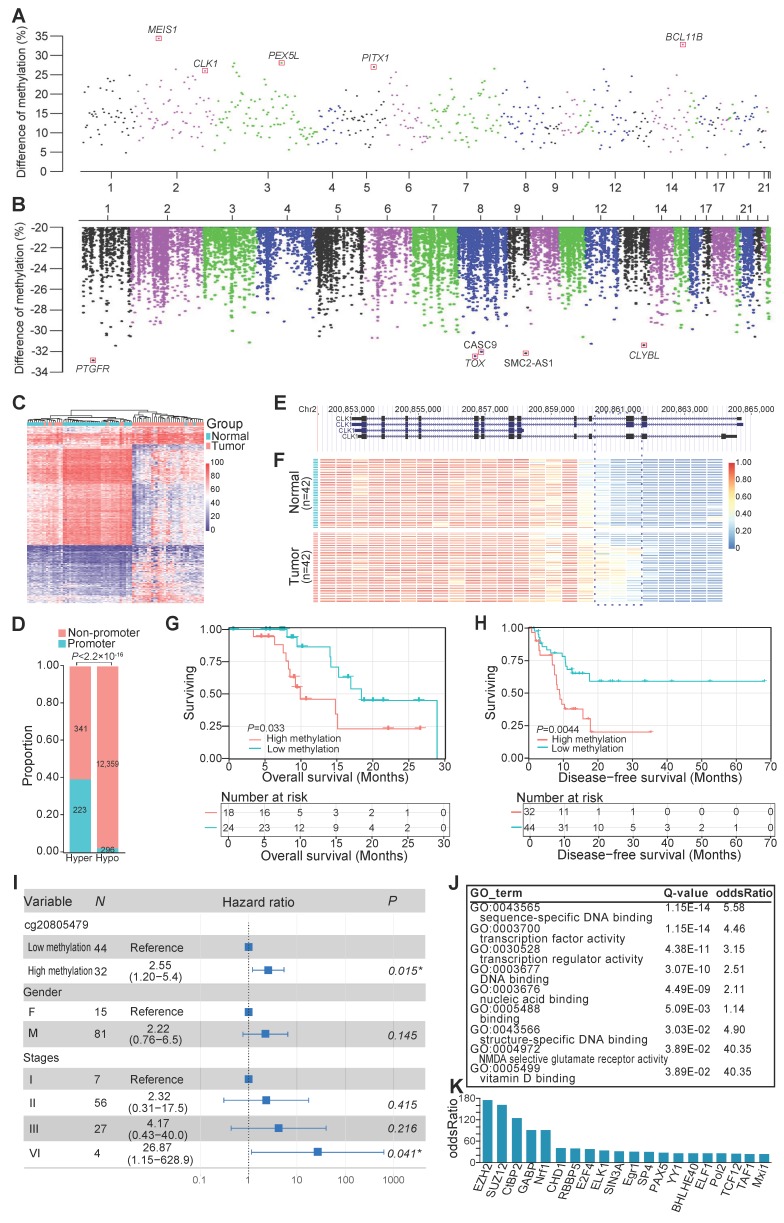
DNA methylation alteration within promoters in ESCC. Genome-wide distribution of significantly hypermethylated (A) and hypomethylated (B) regions in ESCC. (C) Hierarchical clustering for methylation values of aberrantly methylated promoters. (D) Proportion of promoter-associated DMRs to aberrantly hypermethylated (hyper) or hypomethylated (hypo) regions. Example of tumor-specific hypermethylation at the promoter or the intron of *CLK1* transcripts (E, F), one of the strongest genome-wide signals on chromosome 2 (A). Kaplan-Meier plot showing overall survival (G) and disease-free survival (H) stratified by ESCC patients of our sequenced (G) and TCGA-ESCA cohort (H) according to methylation levels of the *CLK1* promoter locus, respectively. (I) Multivariate Cox regression analysis of methylation levels based on a probe (cg20805479) within the *CLK1* promoter locus after controlling for gender and TNM stage. (J) Functional enrichment of genes with differentially hypermethylated promoters in ESCC. (K) Transcription factors binding site enrichment of aberrantly hypermethylated promoter regions.

To dissect the DNAm changes associated with ESCC carcinogenesis, we performed differential DNAm analysis on all matched tumor and normal tissues. Genome-wide DNAm changes between cancer and normal tissues revealed 13,219 differentially methylated regions (DMRs) (**Figure 2A, B**), 95.73% of which were hypomethylated in cancer tissues (**Figure 2B**). In light of the importance of promoter regions in regulating the gene expression, we performed hierarchical cluster analysis based on methylation values of 519 aberrantly methylated promoter regions and observed that it could discriminate the majority of malignant and adjacent tissues (**Figure 2C**). Interestingly, our data showed that 39.54% of 564 hypermethylated regions resided in promoter regions, whereas only 3% of 12,655 hypomethylated regions were identified in the promoter regions (**Figure 2D**). These data indicated that hyper- and hypomethylated changes had distinct genomic distribution, and increased DNAm alteration tended to occur within promoters. For example, one of the strongest tumor-specific hypermethylated signals on chromosome 2 was located in the promoter or the intron of CDC-like kinase 1 (*CLK1*) transcripts (**Figure 2 A, E, F**). The methylation level of the *CLK1* locus was predicted to be negatively correlated with overall survival of the ESCC patients (**Figure 2G**) and disease-free survival of ESCC patients in the TCGA-ESCA cohort (**Figure 2H**), even after adjusting for multiple clinical factors, including gender and TNM staging (**Figure 2I**). To understand the overall functional relevance of genes containing hypermethylated promoters, we performed gene ontology enrichment analysis. Genes related to sequence-specific DNA binding and transcription factor activity were found to be significantly enriched (**Figure 2J**), and some of them were well-known tumor suppressors, such as BCL11B 41 and PITX1 42. Furthermore, transcription factor binding site enrichment analysis for all identified hypermethylated regions revealed 20 significantly enriched transcription factors including several well-established ESCC-associated genes (**Figure 2K**), such as EZH2 43, SUZ12, and CtBP2 44. These findings highlighted the widespread involvement of transcriptional regulators in the pathogenesis of ESCC.

**Figure 3:**
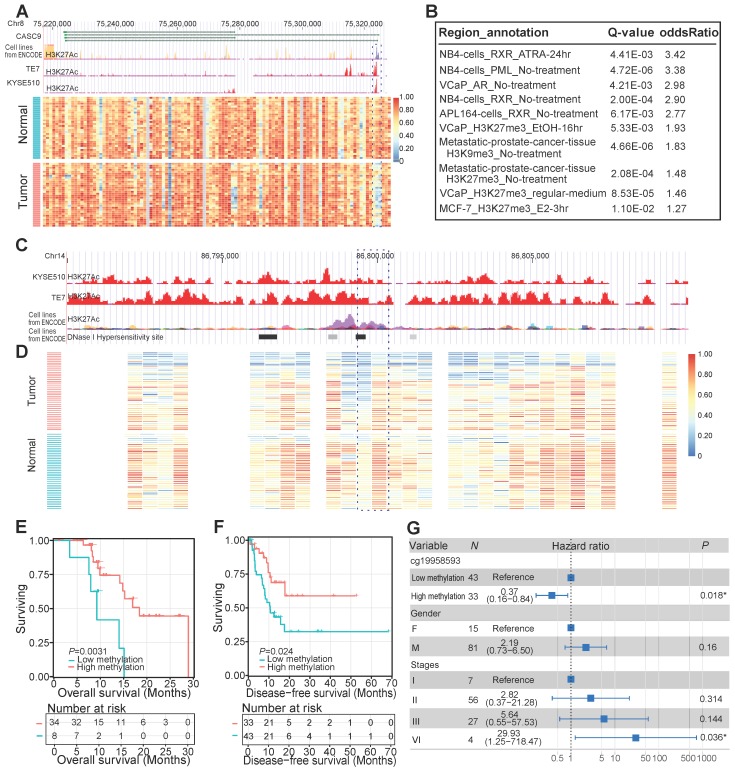
Hypomethylated changes within noncoding regions in ESCC**.** (A) Tumor-specific hypomethylation at the CASC9 promoter locus with decreased DNA methylation in the sequenced ESCC tumors and high histone H3K27 acetylation in ESCC cell lines. H3K27ac profiles include a cross-tissue consensus track from the ENCODE database and ChIP-seq data from two ESCC cell lines (TE7, KYSE510) from a previous study 39. (B) Significant overlap of aberrantly hypomethylated intergenic regions with public annotation data of cancer cell lines, based on LOLA Core enrichment analysis 40. Tumor-specific hypomethylation at the intergenic locus (Chr14: 86,799,804-86,800,434) with decreased DNA methylation in the sequenced ESCC tumors (D) and high histone H3K27 acetylation in ESCC and ENCODE cell lines (C). Kaplan-Meier plots showing overall survival (E) and disease-free survival (F) stratified for the sequenced ESCC patients (E) and the TCGA-ESCA cohort (F) according to methylation levels of the intergenic locus (Chr14: 86,799,804-86,800,434). (G) Multivariate Cox regression analysis based on methylation levels of a probe (cg19958593) within the intergenic locus (Chr14: 86,799,804-86,800,434) after controlling for gender and TNM stage.

As mentioned above, the majority of all identified DMRs were hypomethylated in cancer tissues. One example of such a tumor-specific hypomethylated region was located in the promoter of a well-known ESCC-implicated long noncoding RNA (lncRNA), CASC9 (**Figure 3A**). This tumor-associated lncRNA was reported to function as an oncogene by downregulating the expression of *PDCD4* through recruiting EZH2 and altering trimethylation levels of H3K27 in ESCC cells 45. From the perspective of DNAm, our study highlighted the critical role of CASC9 as a valuable marker for ESCC diagnosis and prognosis 45, 46. Furthermore, we observed that 55.93% of identified hypomethylated regions resided in distal intergenic regions. We performed region set enrichment analysis of these DMRs using LOLA 40 to detect enriched regulators based on collected regulatory region sets of chromatin immunoprecipitation sequencing peaks 47, 48 and DNaseI hypersensitive elements 49. As expected, DMRs located within these intergenic regions were enriched for H3K27me3 modification sites (**Figure 3B**), a mark associated with chromosome inactivation 50. Interestingly, DMRs within these noncoding regions were also heavily enriched for binding sites of retinoid X receptor (RXR) (**Figure 3B**), which co-occupies the active enhancers defined by H3K27ac 51, suggesting the modulating role of RXR during carcinogenesis and development of ESCC. One example of such DMRs was located on Chr14: 86,799,804-86,800,434 (**Figure 3C, D**) surrounded by several lncRNAs, including LINC01148 and LINC02309. Decreased DNAm of this region was observed in ESCC tissues of our sequenced samples (**Figure 3D**). Another observation in ESCC cell lines in this region was of the high level of histone H3K27 acetylation (**Figure 3C**), a chromatin marker associated with gene activation and active enhancers 52. Also, a peak of the DNase I hypersensitivity site, a reflection of chromatin accessibility, was observed in this region in cell lines from the ENCODE database (**Figure 3C**). The methylation levels of this intergenic locus were predicted to be positively correlated with the overall survival of the sequenced ESCC patients (**Figure 3E**). This contention was further validated in ESCC patients from another independent TCGA-ESCA cohort (**Figure 3F**) even after adjusting for multiple clinical factors, including gender and TNM staging (**Figure 3G**). These data, together with other reports 39, 53, 54, suggest a regulatory role for these noncoding regions in ESCC pathogenesis, highlighting the importance of further analysis of these regions.

### DNAm changes associated with LNM of ESCC patients

**Figure 4:**
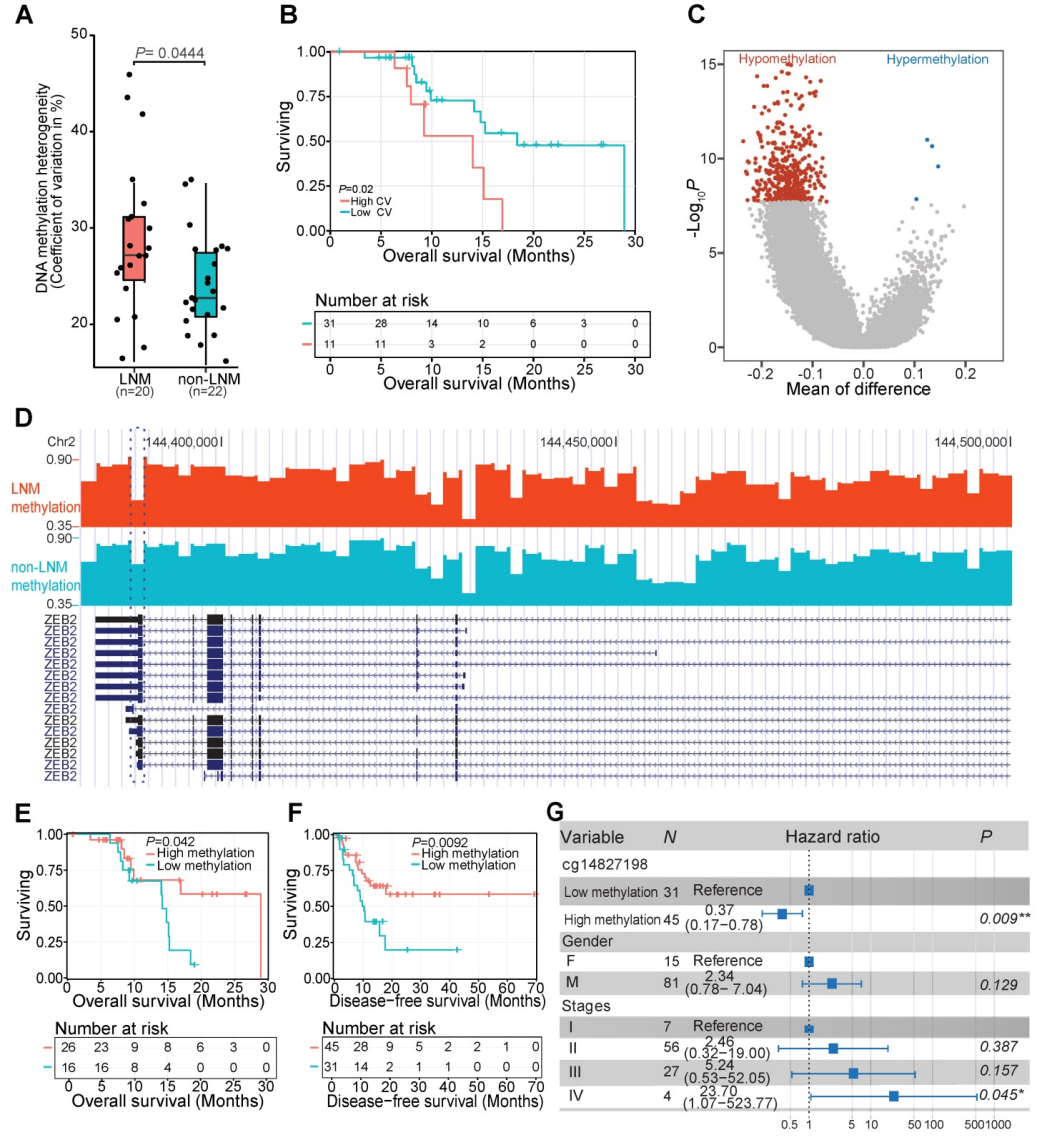
DNA methylation changes associated with LNM in ESCC patients**.** (A) Interindividual DNA methylation heterogeneity quantified by the coefficient of variation (CV) between patients with or without LNM. The coefficient of variation across each genome was calculated as a measure of heterogeneity between samples. (B) Kaplan-Meier plot showing overall survival stratified by subjects from the sequenced ESCC patients according to the interindividual DNA methylation heterogeneity level. (C) Distribution of differentially methylated regions between tissues of patients with or without LNM. Red and blue dots correspond to aberrantly hypomethylated and hypermethylated regions, respectively. (D) LNM-specific hypomethylation at the 3' untranslated region of *ZEB2* in patients with LNM. Kaplan-Meier plots showing overall survival (E) and disease-free survival (F) stratified according to the methylation level of 3' untranslated region of *ZEB2* in the sequenced ESCC patients (E) and TCGA-ESCA cohort (F). (G) Multivariate Cox regression analysis based on the methylation level of a probe (cg14827198) within the 3' untranslated region of* ZEB2* after controlling for gender and TNM stage.

To further identify the relationship between DNAm variation and clinical variables, we explored the association between interindividual DNAm heterogeneity and clinical data. We found that patients with LNM, an early event associated with poor prognosis in ESCC patients 2, 5, exhibited higher interindividual DNAm heterogeneity than patients without LNM (**Figure 4A**). Kaplan-Meier plot showed that ESCC patients with high inter-individual DNAm heterogeneity experienced a worse overall survival (**Figure 4B**). We then compared the methylomes of patients with or without LNM, and identified 490 DMRs associated with LNM features of ESCC (**Figure 4C**) (**Supplementary Table S3**). For example, decreased DNAm in patients with LNM was observed at 3' untranslated region of *ZEB2* (**Figure 4D**). The low methylation levels of this locus were predicted to correlate with worse overall survival of ESCC patients in our sequenced (**Figure 4E**) and TCGA-ESCA cohorts (**Figure 4F**) even after adjusting for multiple clinical factors (**Figure 4G**). It was previously demonstrated that *ZEB2* could promote metastasis of gastric cancer and colorectal cancer, a modulated epithelial-mesenchymal transition of gastric cancer cells, and was associated with poor prognosis of colorectal cancer 55, 56. Identification of these DMRs provides valuable markers associated with LNM status that might help predict ESCC prognosis in patients with LNM.

### Widespread intratumor DNAm heterogeneity in patients with LNM

**Figure 5:**
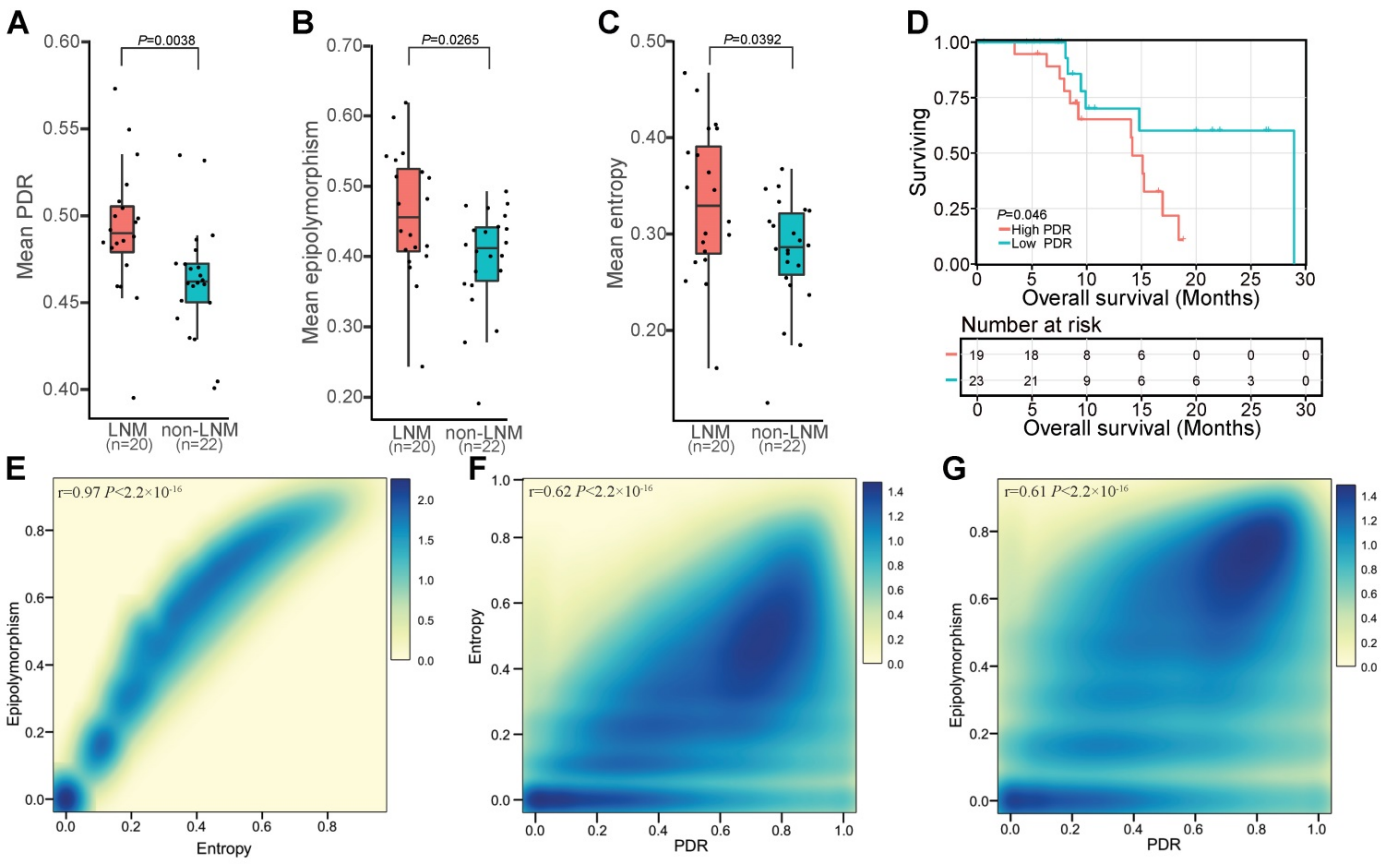
DNA methylation patterns identify widespread intratumor heterogeneity in patients with LNM**.** Distribution of sample-wise discordantly methylated read (PDR) scores (A), epipolymorphism (B), and entropy (C) between patients with or without LNM. (D) Kaplan-Meier plots showing overall survival stratified according to PDR score in subjects from the sequenced patients. Density scatterplot showing the relationship between epipolymorphism and entropy (E), entropy and PDR (F), and epipolymorphism and PDR (G) for 5-kb tiling regions.

In view of the widespread inter-individual DNAm heterogeneity in ESCC, we investigated whether DNAm differences existed between cells within the same tumor. Three methods, PDR, entropy, and epipolymorphism, were used to evaluate intratumor heterogeneity in ESCC. The PDR score is considered to be an indicator of epigenetic instability that might contribute to the clonal selection of individual cells 33, 35. We observed higher locally disordered DNAm heterogeneity in patients with LNM than without LNM (**Figure 5A**). Epipolymorphism is a measurement of the observed consistency of a given methylation pattern within a region versus the expected random pattern and can be used to measure the level of overall epigenetic dysregulation of a specific sample 57-59. Elevated overall epipolymorphism values, reflecting higher heterogeneity, were observed in patients with LNM (**Figure 5B**). Increased sub-clonal variety measured by the epiallele entropy was also observed in patients with LNM (**Figure 5C**). Regions with high epiallele entropy and high epipolymorphism also exhibited higher PDR values, indicating agreement between these methods (**Figure 5E, F, G**) and supporting the conclusion that intratumor DNAm heterogeneity was higher in patients with LNM than in patients without LNM (**Figure 5A, B, C**). To further elucidate the impact of intratumor DNAm heterogeneity on clinical outcome, we performed Kaplan-Meier survival analysis of our sequenced patients stratified according to the PDR score. The PDR level was predicted to correlate with the overall survival of ESCC patients, and patients with a high PDR level exhibited worse overall survival (**Figure 5D**). This finding suggested the prognostic value of intratumor DNAm heterogeneity for ESCC patients.

### Intratumor DNAm heterogeneity within copy number alteration (CNA) regions of ESCC patients

**Figure 6:**
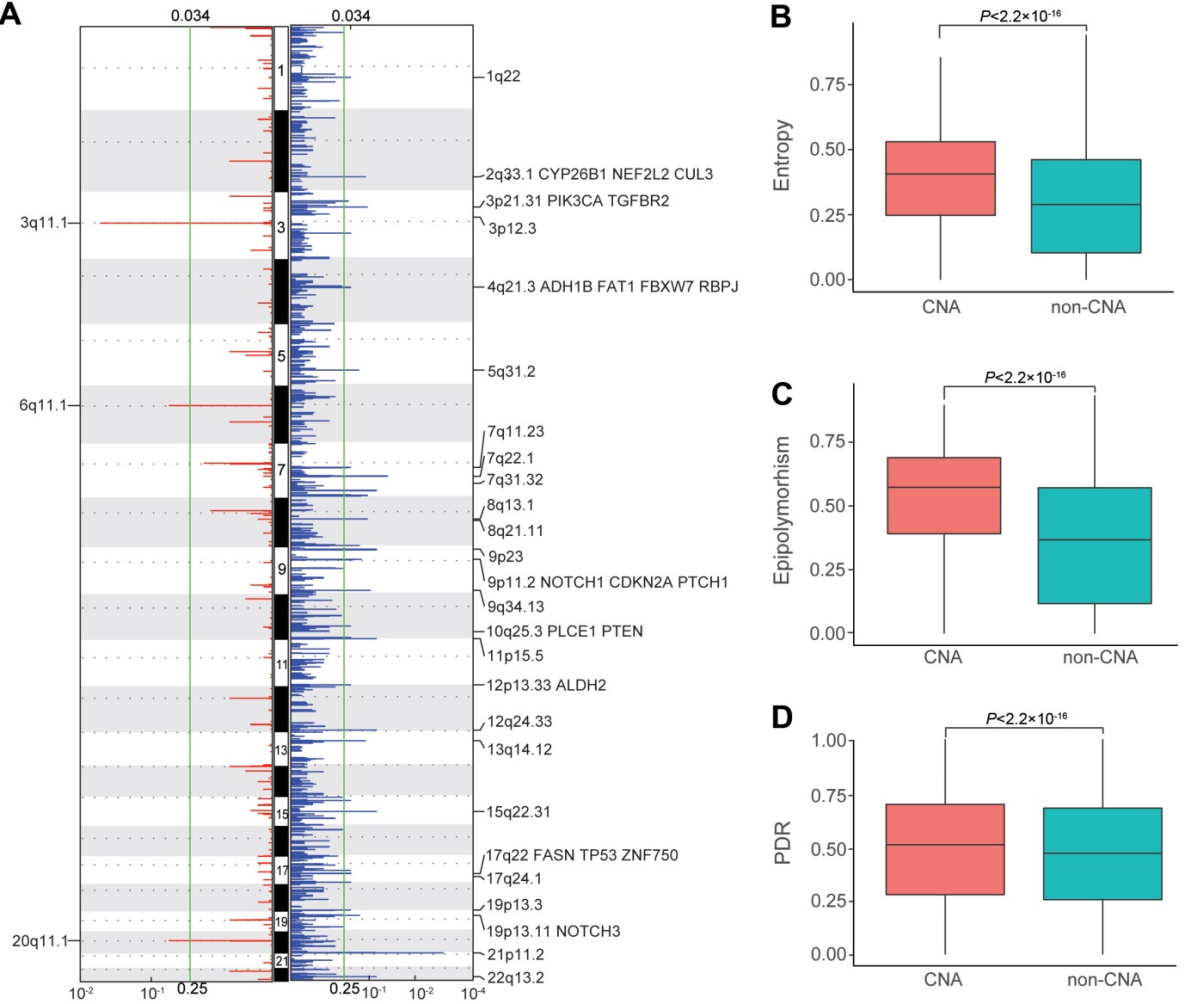
Intratumor DNA methylation heterogeneity within copy number alteration (CNAs) regions of ESCC patients**.** (A) Regions of recurrent focal amplifications (left) and focal deletions (right) are plotted by the false discovery rate (x-axis) for each chromosome (y-axis). Distribution of entropy (B), epipolymorphism (C), and discordantly methylated read (PDR) scores (D) across the genome of ESCC patients between CNA and non-CNA regions

Considering the pivotal role of copy number alterations in the pathogenesis of ESCC 4, 6-10, we compared intratumor DNAm heterogeneity within CNA and non-CNA regions of ESCC patients. We first identified somatic CNAs of each patient from the WGBS data and then analyzed somatic CNA data with Gistic2 to define recurrently amplified and deleted regions. Consistent with previous studies, we detected several well-defined ESCC-associated CNAs (**Figure 6A**), including *CYP26B1* 60, *CUL3* 8, *ADH1B,* and *ALDH2* 61. Tumor tissues had an average entropy level of 0.3812 within CNA regions but displayed a much-decreased level of 0.2924 in the non-CNA regions. Also, CNA regions with higher entropy exhibited higher epipolymorphism and PDR values (**Figure 6B, C, D**). These results revealed that intratumor DNAm heterogeneity varied across genome segments of ESCC patients, and was higher in CNA regions compared to that in non-CNA regions, indicating a close link between epigenetic variations and structural variability.

## Discussion

Inter- and intratumor heterogeneity fuels resistance to therapy in multiple cancers 28. Thus, detailed knowledge of tumor heterogeneity is beneficial for the clinical management of cancer patients. ESCC, one of the leading causes of cancer mortality worldwide and a heterogeneous cancer with diverse clinical manifestations 62, represents an ideal model to investigate tumor heterogeneity and progression. Recently, it has been reported that ESCC displays higher intratumor mutational heterogeneity than several other cancer types, including esophageal adenocarcinoma (EAC), which is another predominant histopathological subtype of esophageal cancer 63. Also, analysis of ESCC by multiple region whole-exome sequencing to analyze intratumor heterogeneity identified several heterogeneous somatic mutations in tumor-suppressor genes such as *TP53*, *ZNF750,* and *KMT2D* 29, 64. A higher intratumor heterogeneity in EAC has been reported to be associated with a poor response to neoadjuvant chemotherapy 30. However, when mutant-allele tumor heterogeneity (MATH) 65 based on whole-exome sequencing data of ESCC patients in the TCGA database was analyzed, no significant association was observed between intratumor heterogeneity and clinical outcomes, including overall survival, progression-free survival, and disease-specific survival (**Supplementary Figure S1**). Thus, comprehensive analysis beyond the level of genetic heterogeneity is required to explain the poor overall 5-year survival rates of ESCC. Herein, using single-base resolution WGBS, we identified population-level DNAm variations in ESCC, which manifested greater inter-individual DNAm heterogeneity than prostate cancer or chronic lymphocytic leukemia, two well-recognized heterogeneous cancer types 35. We also observed increased inter-individual and intra-tumor DNAm heterogeneity in patients with LNM, which is a clinical feature tightly correlated with ESCC patient prognosis 2, 5. This was consistent with the observation that patients with more aggressive disease tend to exhibit high tumor heterogeneity 66. Furthermore, our data demonstrated that ESCC patients with high inter- or intratumor DNAm heterogeneity experienced worse overall survival. Similarly, a high level of DNAm heterogeneity was also observed to be associated with adverse clinical outcomes of chronic lymphocytic leukemia patients 58. Together, these data suggest the prognostic value of inter- and intratumor epi-heterogeneity for ESCC patients, although a larger cohort is still needed to validate these findings.

Consistent with the systematically reduced DNAm level in cancers, we observed that the majority of identified DMRs were hypomethylated in ESCC tissues. Compared with the hypomethylated regions in ESCC, the identified hypermethylated regions were enriched in promoters of transcription factors, including BCL11B and PITX1, two well-known tumor suppressors 41 42. Furthermore, transcription factor binding site enrichment analysis for all identified hypermethylated regions revealed several well-known ESCC-associated genes, including EZH2 43, SUZ12, and CtBP2 44. These data suggested the essential role of transcription factors during the pathogenesis of ESCC.

Interestingly, we observed that more than half of the identified hypomethylated regions in ESCC were located in the distal intergenic regions, which were greatly enriched for binding sites of retinoid X receptor (RXR) that co-occupies active enhancers defined by H3K27ac 51. Regional enrichment of these demethylated regions suggests a correlation between DNA hypomethylation and activation of histone markers in ESCC. This notion is consistent with a previous study showing that DNA hypermethylation in super-enhancers of ESCC reduced active histone markers 39. The methylation levels of several intergenic loci were predicted to be correlated with the overall survival of our sequenced ESCC patients, which was further validated in ESCC patients from another independent cohort, the TCGA-ESCA, even after adjusting for multiple clinical factors, including gender and TNM staging. Additionally, increased lncRNA expression in noncoding regions is reported to promote ESCC cell proliferation, migration, invasion, and growth of xenograft tumors 53. Our data, together with previous reports 39, 53, 54, suggest a regulatory role for these noncoding regions in ESCC pathogenesis, highlighting the importance of further analysis of these regions.

## Conclusions

In summary, we presented high-resolution, population-level DNAm variations in ESCC, provided evidence for widespread intratumor DNAm heterogeneity among ESCC patients, and identified numerous DNAm alterations associated with ESCC carcinogenesis and progression. Our study might facilitate a better prognosis of this prevalent disease and provide a translational basis for designing personalized medicine strategies.

## Supplementary Material

Supplementary figure and tables.

## Abbreviations

ESCC: Esophageal squamous cell carcinoma
DNAm: DNA methylation
LNM: lymph node metastasis
CNAs: copy number alterations
TCGA: The Cancer Genome Atlas
PDR: discordantly methylated reads
WGBS: whole-genome bisulfite sequencing
DMRs: differentially methylated regions
MDS: multidimensional scaling
CV: coefficient of variation
CLL: chronic lymphocytic leukemia
CLK1: CDC like kinase 1
