# Supplementary material for: Inter- and intratumor DNA methylation heterogeneity associated with lymph node metastasis and prognosis of esophageal squamous cell carcinoma
Source: Theranostics. 2020 Feb 10;10(7):3035–48. doi: 10.7150/thno.42559 (PMC7053185; doi:10.7150/thno.42559)
Supplement: Supplementary file 1 — Supplementary figure and tables. [file thnov10p3035s1.pdf]

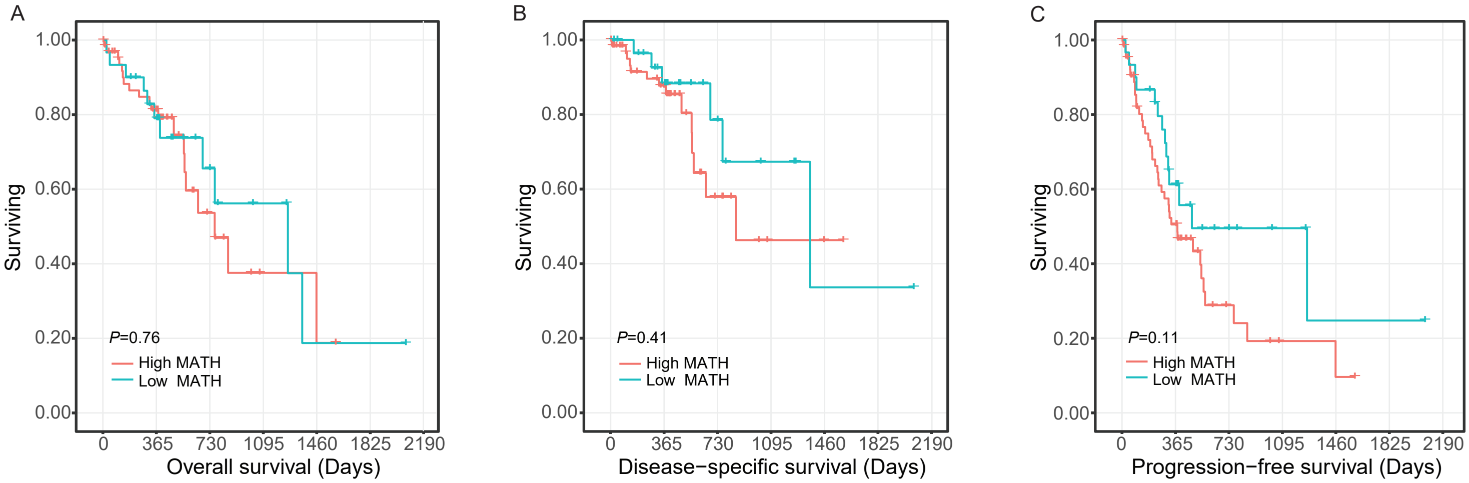

Supplementary Figure S1

Table S1 Clinical data of sequenced esophageal squamous cell carcinoma patients

Sample ID	Disease type	Patient ID	Tissue type	Sex	Date of surgery	Last follow-up time	Vital status	Age	Pathological morphology	TNM stage	Number of examined lymph node	Number of positive lymph node	Lymph node metastasis	Number of reads	Number of mapped bases	Mean coverage (×)
s_99			Noncancerous tissue											627,080,746	87,805,829,706	27.36
s_100	Esophageal Squamous Cell Carcinoma	ESCC99100	Tumor tissue	Male	2009-7-2	2010-2-9	Alive	75	Constritive	II-III	18	1	Yes	528,512,847	74,295,142,193	23.15
s_101			Noncancerous tissue											583,277,532	80,028,690,329	24.94
s_102	Esophageal Squamous Cell Carcinoma	ESCC101102	Tumor tissue	Male	2009-9-7	2010-2-9	Alive	60	Ulcerative	I	24	0	No	573,192,997	79,490,913,713	24.77
s_104			Noncancerous tissue											715,158,371	98,895,858,619	30.82
s_103	Esophageal Squamous Cell Carcinoma	ESCC103104	Tumor tissue	Male	2010-11-24	2013-1-24	Alive	56	Ulcerative	II	33	2	Yes	563,655,474	78,299,032,712	24.40
s_106			Noncancerous tissue											587,240,157	81,234,936,174	25.31
s_105	Esophageal Squamous Cell Carcinoma	ESCC105106	Tumor tissue	Male	2010-11-18	2013-1-24	Alive	72	Protuberant	II	18	0	No	609,699,113	85,252,635,269	26.56
s_11			Noncancerous tissue											669,146,113	92,885,665,360	28.94
s_12	Esophageal Squamous Cell Carcinoma	ESCC1112	Tumor tissue	Male	2006-8-28	2008-1-7	Alive	69	Borrmann	II-III	17	0	No	581,949,674	80,943,018,448	25.22
s_17			Noncancerous tissue											581,580,318	80,706,439,178	25.15
s_18	Esophageal Squamous Cell Carcinoma	ESCC1718	Tumor tissue	Male	2006-3-7	2006-12-9	Dead	56	Protuberant	II	17	2	Yes	668,573,748	93,254,037,281	29.06
s_19			Noncancerous tissue											534,200,765	73,488,388,173	22.90

s_20	Esophageal Squamous Cell Carcinoma	ESCC1920	Tumor tissue	Male	2006-3-10	2007-5-9	Dead	51	Ulcerative	II	4	0	No	588,232,343	81,862,734,719	25.51
s_21			Noncancerous tissue											531,837,755	74,657,039,902	23.26
s_22	Esophageal Squamous Cell Carcinoma	ESCC2122	Tumor tissue	Male	2006-3-13	2008-1-7	Alive	51	Protuberant	II	10	0	No	525,968,655	73,112,844,952	22.78
s_25			Noncancerous tissue											578,114,144	79,677,613,876	24.83
s_26	Esophageal Squamous Cell Carcinoma	ESCC2526	Tumor tissue	Male	2006-3-17	2008-8-1	Dead	58	Ulcerative	III	7	0	No	429,511,252	60,402,136,525	18.82
s_27			Noncancerous tissue											923,124,902	127,510,372,417	39.73
s_28	Esophageal Squamous Cell Carcinoma	ESCC2728	Tumor tissue	Female	2006-4-3	2008-1-7	Alive	37	Protuberant	II	5	0	No	517,941,838	71,646,227,410	22.32
s_29			Noncancerous tissue											527,126,017	72,842,812,701	22.70
s_30	Esophageal Squamous Cell Carcinoma	ESCC2930	Tumor tissue	Female	2006-4-21	2007-7-9	Dead	67	Ulcerative	III	17	4	Yes	558,928,945	77,807,112,914	24.24
s_31			Noncancerous tissue											688,352,647	96,709,214,502	30.13
s_32	Esophageal Squamous Cell Carcinoma	ESCC3132	Tumor tissue	Male	2006-4-28	2007-11-1	Dead	60		II	3	1	Yes	532,135,191	74,055,269,811	23.08
s_33			Noncancerous tissue											540,103,639	74,934,868,324	23.35
s_34	Esophageal Squamous Cell Carcinoma	ESCC3334	Tumor tissue	Male	2006-5-18	2008-1-8	Alive	56	Plaque	II	19	2	Yes	668,175,115	92,322,799,609	28.77
s_35			Noncancerous tissue											477,990,867	66,351,662,476	20.67
s_36	Esophageal Squamous Cell Carcinoma	ESCC3536	Tumor tissue	Male	2006-5-22	2007-3-15	Dead	64	Ulcerative	I-II	3	0	No	648,644,970	90,589,699,053	28.23

s_37			Noncancerous tissue											555,231,373	77,020,362,782	24.00
s_38	Esophageal Squamous Cell Carcinoma	ESCC3738	Tumor tissue	Female	2006-6-22	2008-1-7	Alive	36	Ulcerative	II	1	0	No	826,774,842	114,300,768,420	35.62
s_39			Noncancerous tissue											497,615,062	68,540,823,114	21.36
s_40	Esophageal Squamous Cell Carcinoma	ESCC3940	Tumor tissue	Male	2006-6-28	2007-3-9	Dead	59	Ulcerative	II	12	0	No	463,814,164	64,117,948,266	19.98
s_41			Noncancerous tissue											543,026,843	75,502,056,014	23.53
s_42	Esophageal Squamous Cell Carcinoma	ESCC4142	Tumor tissue	Male	2006-11-27	2007-3-9	Dead	73	Protuberant	II	15	0	No	542,465,075	75,147,678,114	23.42
s_43			Noncancerous tissue											457,522,370	63,876,874,020	19.90
s_44	Esophageal Squamous Cell Carcinoma	ESCC4344	Tumor tissue	Male	2007-11-2	2008-7-1	Dead	65	Protuberant	II	26	0	No	550,287,399	76,568,630,928	23.86
s_45			Noncancerous tissue											577,046,333	79,708,809,707	24.84
s_46	Esophageal Squamous Cell Carcinoma	ESCC4546	Tumor tissue	Female	2007-11-28	2008-7-10	Alive	70	Fungoid	II	12	1	Yes	562,418,947	78,160,445,578	24.35
s_49			Noncancerous tissue											801,898,125	110,465,134,380	34.42
s_50	Esophageal Squamous Cell Carcinoma	ESCC4950	Tumor tissue	Female	2007-9-24	2008-4-1	Dead	55	Ulcerative	I	22	4	Yes	562,618,161	77,568,334,061	24.17
s_51			Noncancerous tissue											596,688,754	82,350,855,135	25.66
s_52	Esophageal Squamous Cell Carcinoma	ESCC5152	Tumor tissue	Female	2007-8-28	2008-7-15	Alive	57		II	11	0	No	587,226,199	81,411,096,331	25.37
s_53			Noncancerous tissue											574,577,877	79,515,649,146	24.78

s_54	Esophageal Squamous Cell Carcinoma	ESCC5354	Tumor tissue	Male	2008-5-19	2008-12-24	Alive	57	Borrmann	I			No	699,143,803	96,703,211,339	30.13
s_57			Noncancerous tissue											699,052,222	97,362,458,773	30.34
s_58	Esophageal Squamous Cell Carcinoma	ESCC5758	Tumor tissue	Male	2007-9-6	2008-12-6	Dead	78	Ulcerative	II-III	3	0	No	601,942,489	83,931,059,710	26.15
s_59			Noncancerous tissue											651,362,295	90,562,826,644	28.22
s_60	Esophageal Squamous Cell Carcinoma	ESCC5960	Tumor tissue	Male	2007-10-16	2008-7-10	Alive	57	Constritive	II	6	1	Yes	605,937,307	84,373,570,297	26.29
s_61			Noncancerous tissue											595,689,298	82,780,059,627	25.79
s_62	Esophageal Squamous Cell Carcinoma	ESCC6162	Tumor tissue	Male	2007-10-23	2008-7-10	Alive	53	Protuberant	I-II	36	0	No	638,489,824	88,693,004,471	27.64
s_65			Noncancerous tissue											576,923,214	79,164,947,900	24.67
s_66	Esophageal Squamous Cell Carcinoma	ESCC6566	Tumor tissue	Male	2008-7-4	2009-3-9	Dead	58		II	30	2	Yes	599,989,722	83,117,147,923	25.90
s_67			Noncancerous tissue											597,981,755	82,557,142,686	25.72
s_68	Esophageal Squamous Cell Carcinoma	ESCC6768	Tumor tissue	Male	2008-7-8	2008-12-26	Alive	72	Peviform	II	23	0	No	580,488,194	81,756,577,600	25.48
s_69			Noncancerous tissue											481,478,264	66,800,127,957	20.81
s_70	Esophageal Squamous Cell Carcinoma	ESCC6970	Tumor tissue	Male	2008-7-14	2008-12-26	Alive	59	Borrmann	II	24	3	Yes	545,301,499	77,295,114,796	24.08
s_71			Noncancerous tissue											551,872,361	76,507,382,001	23.84
s_72	Esophageal Squamous Cell Carcinoma	ESCC7172	Tumor tissue	Male	2008-8-6	2009-4-1	Dead	59	Ulcerative	II	19	8	Yes	923,261,788	127,623,435,531	39.77

s_73			Noncancerous tissue											750,919,668	104,776,483,926	32.65
s_74	Esophageal Squamous Cell Carcinoma	ESCC7374	Tumor tissue	Male	2008-3-5	2009-6-1	Dead	56	Ulcerative	II	34	1	Yes	855,725,289	118,843,411,730	37.03
s_75			Noncancerous tissue											391,157,702	54,694,107,711	17.04
s_76	Esophageal Squamous Cell Carcinoma	ESCC7576	Tumor tissue	Male	2008-6-11	2009-11-1	Dead	53	Ulcerative	I-II	30	0	No	525,756,355	72,472,500,782	22.58
s_77			Noncancerous tissue											594,406,235	81,885,817,702	25.52
s_78	Esophageal Squamous Cell Carcinoma	ESCC7778	Tumor tissue	Female	2008-12-5	2008-12-23	Alive	56	Ulcerative	I-II	21	0	No	604,835,098	84,260,245,567	26.26
s_79			Noncancerous tissue											564,467,042	78,035,937,947	24.32
s_80	Esophageal Squamous Cell Carcinoma	ESCC7980	Tumor tissue	Female	2008-8-11	2008-12-24	Alive	59	Ulcerative	II	32	0	No	549,956,529	76,837,224,129	23.94
s_81			Noncancerous tissue											558,064,591	77,157,318,216	24.04
s_82	Esophageal Squamous Cell Carcinoma	ESCC8182	Tumor tissue	Female	2008-12-15	2010-2-9	Dead	62	Ulcerative	II	19	1	Yes	563,731,146	78,500,981,312	24.46
s_83			Noncancerous tissue											624,931,596	86,929,354,312	27.09
s_84	Esophageal Squamous Cell Carcinoma	ESCC8384	Tumor tissue	Male	2007-2-28	2007-12-9	Dead	59	Ulcerative	II	3	3	Yes	578,495,566	80,347,661,211	25.04
s_85			Noncancerous tissue											547,018,517	75,755,384,648	23.61
s_86	Esophageal Squamous Cell Carcinoma	ESCC8586	Tumor tissue	Female	2007-3-7	2008-1-7	Alive	58	Ulcerative	II	17	0	No	529,134,469	73,892,050,017	23.02
s_87			Noncancerous tissue											669,892,707	93,252,586,066	29.06

s_88	Esophageal Squamous Cell Carcinoma	ESCC8788	Tumor tissue	Female	2007-3-29	2008-1-8	Alive	58	Borrmann	II	23	3	Yes	573,809,593	79,414,043,038	24.75
s_89			Noncancerous tissue											766,151,296	106,325,726,688	33.13
s_90	Esophageal Squamous Cell Carcinoma	ESCC8990	Tumor tissue	Male	2007-4-11	2008-1-7	Alive	50	Ulcerative	II-III	13	7	Yes	467,741,038	64,712,861,801	20.16
s_91			Noncancerous tissue											646,870,160	89,302,712,605	27.83
s_92	Esophageal Squamous Cell Carcinoma	ESCC9192	Tumor tissue	Male	2007-5-28	2008-1-9	Dead	70	Ulcerative	II	1	0	No	518,965,863	72,743,335,517	22.67
s_93			Noncancerous tissue											751,973,966	104,690,776,124	32.62
s_94	Esophageal Squamous Cell Carcinoma	ESCC9394	Tumor tissue	Male	2009-5-11	2010-2-9	Alive	52	Ulcerative	II	16	1	Yes	773,608,982	107,624,745,850	33.54
s_95			Noncancerous tissue											575,347,961	79,911,388,520	24.90
s_96	Esophageal Squamous Cell Carcinoma	ESCC9596	Tumor tissue	Female	2009-6-23	2010-2-9	Alive	50	Ulcerative	II	35	1	Yes	640,856,211	89,083,002,606	27.76
s_97			Noncancerous tissue											624,015,277	86,796,635,553	27.05
s_98	Esophageal Squamous Cell Carcinoma	ESCC9798	Tumor tissue	Male	2009-7-10	2010-2-9	Alive	83	Ulcerative	II	15	3	Yes	525,122,718	74,330,704,862	23.16

Table S2 Mutation hotspots panel

TP53	CDKN2A	RB1	CCND1	NFE2L2	KEAP1	EP300	CREBBP
KMT2D	KMT2C (M	KDM6A	NSD1	YAP1	FAT1	FAT2	FAT3
AJUBA	NOTCH1	NOTCH2	NOTCH3	FBXW7	PIK3CA	EGFR	PTCH1
BRCA2	APC	BRCA1	RNF213	MLH1	ADAM29	FAM135B	SETD1B

Table S3 DNA methylation changes associated with lymph node metastasis of ESCC patients

Chr	Start	End	Qvalue	Mean of difference	Number of CpGs	Methylation level of non-LNM patients	Methylation level of patients with LNM
chr1	49728600	49730178	0.0012	14.722045	15	44.607	29.885
chr1	69843112	69844675	0.0026	12.996775	24	61.146	48.15
chr1	125074394	125075818	0.00092	9.851288	49	58.916	49.065
chr1	125080197	125083395	1.20E-08	11.276868	76	58.561	47.284
chr1	125152703	125154632	3.20E-05	11.554577	50	58.448	46.893
chr1	125162862	125166604	0.027	8.191413	130	63.108	54.844
chr1	143224235	143226166	1.90E-08	8.402629	89	66.308	57.906
chr1	158999079	158999806	6.20E-05	14.224782	18	38.866	24.641
chr1	176913620	176915267	0.031	14.403095	20	59.703	45.3
chr1	182228245	182228955	3.20E-11	17.608968	20	59.547	41.938
chr1	193578596	193579401	0.0062	14.662067	18	77.212	62.55
chr1	207692088	207692432	0.035	16.942897	12	52.621	35.678
chr1	213863242	213863988	0.045	16.655542	15	60.619	43.963
chr1	247861126	247862426	0.0026	17.288549	17	54.623	37.335
chr2	2383016	2384095	0.041	14.827813	15	56.858	42.03
chr2	4718689	4719969	0.035	17.782025	13	56.587	38.805
chr2	5252755	5254909	0.0079	12.917404	32	61.956	49.038
chr2	6263387	6265058	8.10E-05	12.395866	21	55.273	42.878
chr2	21767308	21770063	3.20E-05	13.780518	31	55.31	41.529
chr2	22011635	22013097	0.027	11.555789	21	45.191	33.635
chr2	22051026	22052752	0.016	14.401475	16	53.929	39.527
chr2	22424833	22426838	0.014	12.386854	24	52.221	39.834
chr2	40358952	40359339	0.0014	15.467346	10	49.268	33.801
chr2	49148260	49149040	0.021	16.110492	13	59.5	43.389
chr2	50447962	50449414	0.00087	11.073607	27	47.067	35.994
chr2	58841145	58843233	0.039	14.495925	23	60.728	46.232
chr2	59077419	59079226	0.015	11.576438	26	56.291	44.715
chr2	59739323	59740864	0.017	13.443105	22	67.207	53.764
chr2	76575282	76577088	0.022	12.028562	26	60.885	48.856
chr2	77338245	77340680	0.031	10.170819	24	48.96	38.789
chr2	77368439	77369664	0.0093	15.061222	18	60.898	45.837
chr2	77513051	77514226	0.00075	14.919569	20	51.36	36.441
chr2	77819803	77821582	0.021	11.718842	26	52.79	41.071
chr2	78721514	78721840	0.0033	15.223475	13	50.13	34.907
chr2	80121044	80122199	0.0012	16.053003	16	58.105	42.052
chr2	80209538	80210902	8.80E-06	17.720875	19	59.225	41.504
chr2	80350798	80352420	4.20E-10	19.035853	21	48.186	29.15
chr2	80534639	80535398	0.0061	20.516368	10	63.752	43.236
chr2	80601856	80603434	0.023	14.546991	19	51.518	36.971
chr2	80615287	80616586	0.0093	16.665862	14	51.599	34.933
chr2	80878145	80880339	8.30E-11	16.024809	37	63.088	47.064
chr2	81160407	81162043	0.002	14.358199	24	47.29	32.932
chr2	82577281	82578736	0.0019	13.508718	37	69.53	56.021
chr2	89775171	89777544	1.50E-16	13.726816	108	61.2	50.508
chr2	89777544	89799449	7.60E-22	12.115809	890	59.404	48.539
chr2	89799461	89803370	6.10E-37	12.763064	163	58.735	45.972
chr2	89806034	89807295	0.022	13.30978	47	62.112	49.264
chr2	89809663	89813458	5.10E-05	9.59533	148	62.046	53.302
chr2	89816206	89819328	3.80E-13	11.195915	73	53.874	42.678
chr2	89823790	89826246	1.60E-07	12.667237	51	49.42	35.99
chr2	89826269	89830909	8.10E-20	11.693566	72	44.655	32.961
chr2	89831258	89836943	4.00E-26	10.932784	81	42.165	31.232
chr2	89837262	89841508	5.50E-17	10.970514	62	40.62	29.65
chr2	91410391	91416284	1.30E-45	11.76532	207	57.444	45.678

chr2	91420653	91421528	0.001	10.177648	52	58.974	48.796
chr2	91442791	91443136	5.60E-05	-13.448178	28	35.811	49.259
chr2	116068745	116070019	0.037	14.487828	20	57.277	42.789
chr2	116187879	116189486	0.019	11.89011	28	56.419	44.529
chr2	117152651	117153636	0.0066	17.812325	20	55.783	37.97
chr2	117223420	117223562	0.011	18.398937	11	75.943	57.544
chr2	117253513	117254826	6.20E-05	15.050926	22	51.212	36.162
chr2	117256971	117258566	0.011	15.936802	19	52.903	36.966
chr2	124860877	124862510	0.0045	15.120759	21	61.611	46.491
chr2	124965350	124966421	0.00099	15.97535	19	61.193	45.218
chr2	125203046	125204904	0.00078	13.829469	20	46.975	33.146
chr2	144389317	144389664	0.00027	15.131494	15	63.507	48.375
chr2	165100317	165101515	0.033	13.034911	27	61.542	48.508
chr2	198094922	198095914	0.025	13.252588	16	56.969	43.716
chr2	198137205	198137519	0.0024	16.333447	14	61.593	45.259
chr2	199461076	199461919	2.60E-05	-12.474595	30	42.461	54.936
chr2	209789402	209789844	0.00023	19.433917	13	66.648	47.214
chr3	782537	783679	0.049	16.228806	12	59.232	43.003
chr3	927207	928617	0.00011	14.683741	23	56.559	41.876
chr3	950785	951475	0.00066	16.322467	14	63.821	47.498
chr3	1243531	1244599	0.0019	14.573086	23	71.045	56.472
chr3	1320770	1322352	0.027	12.90054	21	53.337	40.436
chr3	1386022	1386903	0.047	17.758019	10	62.359	44.601
chr3	1519054	1520583	0.012	13.455378	19	53.435	39.979
chr3	2443470	2444543	1.40E-05	20.477776	15	63.062	42.585
chr3	6067195	6067735	0.008	15.367339	18	61.934	46.567
chr3	6085661	6088243	0.045	12.243013	26	59.094	46.851
chr3	6243864	6244818	0.017	17.174955	14	63.527	46.352
chr3	6310211	6311534	0.0014	16.868824	14	62.666	45.797
chr3	6397920	6398870	2.10E-05	20.275662	17	66.855	46.579
chr3	6400331	6401736	0.0026	15.727851	18	72.315	56.587
chr3	6673180	6673682	0.0004	22.505279	12	69.541	47.036
chr3	6761800	6763040	0.024	13.974407	20	59.257	45.282
chr3	6949151	6950018	0.042	16.567768	12	65.044	48.476
chr3	7131854	7133128	0.00058	17.985122	13	61.122	43.137
chr3	7378538	7379414	0.0029	17.373027	10	69.164	51.79
chr3	7415040	7417326	3.90E-06	15.884884	22	66.867	50.982
chr3	8177314	8178921	0.047	13.073116	21	66.975	53.902
chr3	12064461	12065658	0.039	17.934329	10	63.911	45.977
chr3	19235080	19235989	0.0017	18.479834	10	69.139	50.659
chr3	19364199	19364905	0.0011	18.577743	15	67.425	48.847
chr3	19513885	19514149	0.0011	20.517478	10	72.406	51.889
chr3	19634919	19635710	0.011	20.350519	11	57.813	37.462
chr3	35878524	35880045	0.0024	14.213891	26	63.593	49.379
chr3	70300801	70302316	0.012	13.734364	19	67.411	53.677
chr3	80440732	80440983	0.04	15.347245	12	62.828	47.481
chr3	103227409	103229192	0.0092	11.960976	29	48.156	36.195
chr3	116350406	116351821	0.021	14.776587	15	52.326	37.55
chr3	116554219	116554560	0.042	18.173148	12	69.996	51.823
chr3	135411725	135412304	0.018	17.301422	12	62.08	44.778
chr3	147361188	147361446	0.036	-10.361062	16	15.044	25.405
chr3	149967607	149969216	0.0014	18.324299	26	60.168	41.844
chr3	151926428	151927295	0.011	12.665971	19	39.507	26.841
chr3	152026037	152026232	0.00083	15.15425	10	45.235	30.081
chr3	161791096	161791686	0.0072	14.826522	17	61.956	47.129
chr3	164024196	164025924	0.00064	13.605134	39	56.579	42.974
chr3	164553569	164553934	0.04	21.215461	10	61.078	39.862
chr3	164717295	164718744	1.20E-06	15.386085	30	58.174	42.788

chr3	167186827	167187999	0.00027	17.151566	18	53.291	36.139
chr3	174156113	174156490	0.0038	13.595124	20	46.902	33.307
chr3	176719138	176719981	0.037	12.777789	16	46.54	33.762
chr3	181439368	181442294	0.0014	11.860235	37	54.209	42.348
chr3	182596644	182598072	7.30E-05	16.885513	29	67.443	50.558
chr3	190182607	190182809	1.40E-05	25.043796	13	74.054	49.01
chr3	191512862	191517506	0.039	10.371455	55	65.832	55.46
chr3	196024083	196024423	0.0006	17.055784	13	50.065	33.009
chr4	42795382	42796601	1.00E-05	15.654786	23	59.495	43.841
chr4	49113855	49116432	0.00041	9.358137	32	51.211	41.853
chr4	49141519	49144565	0.0084	7.815257	48	49.338	41.523
chr4	49145196	49147228	0.00078	10.476382	28	52.342	41.865
chr4	92026150	92027755	0.039	11.57217	33	69.349	57.776
chr4	107380843	107381575	0.045	12.195388	18	66.559	54.363
chr4	171044485	171045249	8.00E-05	16.345363	12	58.82	42.475
chr4	173763773	173764513	0.01	14.49729	22	70.217	55.72
chr5	390100	390639	0.00067	16.774602	22	67.093	50.318
chr5	5637861	5639205	0.0031	16.199436	15	50.214	34.015
chr5	5680748	5681447	0.024	16.17862	10	57.363	41.184
chr5	6824052	6825160	0.00013	17.818175	17	60.422	42.604
chr5	8294009	8294438	0.015	15.266737	13	52.864	37.598
chr5	8389134	8389505	0.0054	17.934715	11	57.568	39.634
chr5	8486827	8487965	0.041	15.488272	16	57.79	42.301
chr5	8558305	8558660	0.0052	12.046391	10	40.608	28.562
chr5	8709067	8711649	0.017	12.097757	32	49.777	37.68
chr5	11075868	11077893	0.039	12.063567	24	49.026	36.962
chr5	11760537	11761240	0.014	16.17476	16	51.825	35.65
chr5	11794007	11795571	0.0052	12.472382	26	51.114	38.641
chr5	11885616	11886166	0.021	15.943181	10	47.952	32.009
chr5	11892657	11894396	0.0075	13.703823	26	52.212	38.509
chr5	12483209	12483917	0.00063	15.734566	16	57.255	41.52
chr5	12490229	12490742	0.0022	16.13259	19	60.353	44.22
chr5	16128908	16130352	0.019	14.149205	19	40.637	26.488
chr5	16223770	16225407	6.30E-05	13.265147	27	47.532	34.267
chr5	35404435	35405361	2.70E-07	16.806088	17	61.274	44.468
chr5	44371195	44372189	0.012	16.631631	16	48.604	31.972
chr5	44409862	44411843	0.00048	13.085765	25	51.925	38.839
chr5	44422312	44423561	9.80E-05	15.610435	19	50.209	34.599
chr5	45085500	45086366	0.00034	17.643252	18	59.488	41.845
chr5	96170514	96171853	1.30E-08	17.225593	30	80.848	63.622
chr5	115930203	115930759	3.70E-08	18.906934	18	65.792	46.885
chr5	161514111	161515900	4.80E-07	19.359718	18	58.213	38.853
chr5	161529276	161530094	0.043	20.647899	10	68.136	47.489
chr5	161536635	161538603	0.00017	13.985338	28	63.858	49.872
chr5	162279316	162280758	7.40E-08	14.9234	34	65.114	50.19
chr5	162907573	162908188	0.044	15.788161	15	64.95	49.162
chr5	163276342	163277107	0.0003	15.898113	12	54.121	38.223
chr5	165806645	165809135	0.021	12.148061	28	57.857	45.709
chr5	165894654	165896756	0.016	11.825647	27	61.256	49.43
chr5	167042084	167044358	0.0061	13.639889	30	65.777	52.137
chr6	12755741	12757741	9.70E-06	13.970345	32	52.483	38.513
chr6	38176983	38177457	0.046	14.285064	16	66.728	52.443
chr6	61685339	61686329	0.029	13.329296	13	49.41	36.081
chr6	62790797	62792444	1.50E-05	12.526063	23	50.086	37.56
chr6	63285624	63286024	0.0084	16.992314	12	58.832	41.84
chr6	65106143	65107219	0.00048	13.488101	20	51.212	37.724
chr6	67969089	67969829	0.00055	14.211048	16	55.04	40.829
chr6	81348961	81350630	0.014	14.163914	19	54.703	40.539

chr6	86297382	86298251	0.0022	15.292665	13	55.311	40.018
chr6	98391752	98392360	0.0034	18.523172	10	68.129	49.606
chr6	164661704	164662486	0.043	16.470835	12	53.437	36.966
chr6	166541604	166542129	0.017	16.513532	17	44.686	28.172
chr7	1966772	1966868	0.0013	8.42262	14	84.121	75.698
chr7	8490590	8492249	0.00031	15.168799	28	57.467	42.299
chr7	8594604	8595991	0.046	13.201247	25	53.542	40.341
chr7	8865423	8866920	0.0057	14.966805	20	58.639	43.673
chr7	8991368	8992531	0.033	13.110418	22	56.788	43.678
chr7	9234743	9236784	0.02	12.514192	24	53.919	41.405
chr7	9546697	9546874	0.0043	18.299466	12	73.511	55.211
chr7	14509550	14509779	0.0058	19.126125	10	67.01	47.884
chr7	31638191	31639266	0.028	14.224524	15	48.548	34.324
chr7	37788226	37789443	0.0035	20.036645	17	58.988	38.952
chr7	51871390	51873172	0.038	13.143028	27	59.319	46.176
chr7	52098535	52100207	0.0011	12.440132	24	50.894	38.454
chr7	52768310	52769722	0.0028	15.517488	15	62.635	47.118
chr7	52792336	52793420	0.00032	13.435461	23	59.799	46.364
chr7	53193106	53195406	0.0043	13.034317	30	53.773	40.739
chr7	53320371	53322049	0.0094	14.272145	25	59.373	45.101
chr7	53867604	53869028	0.012	12.184357	24	58.294	46.109
chr7	57173251	57174950	0.0003	17.030549	21	58.445	41.414
chr7	58037164	58044842	1.10E-05	13.265141	228	56.836	45.379
chr7	58048618	58050488	1.60E-09	13.38117	57	54.057	40.676
chr7	58060111	58068054	0.00023	11.524267	212	58.544	47.752
chr7	58116789	58118405	2.20E-11	14.163447	48	58.132	43.969
chr7	60911783	60915271	9.60E-06	10.129716	104	60.413	51.161
chr7	60915400	60917066	9.70E-10	12.239917	60	58.534	46.294
chr7	60919718	60923503	3.30E-05	10.345451	132	56.89	46.176
chr7	60934537	60937016	4.70E-11	11.840618	70	58.515	46.674
chr7	61021351	61026970	1.40E-20	11.37966	146	57.361	45.982
chr7	61027284	61030953	0.00027	11.974644	110	57.892	47.013
chr7	61032238	61035570	5.20E-05	11.744817	123	61.585	51.47
chr7	61039980	61041463	5.90E-07	12.447114	38	54.659	42.212
chr7	61058221	61059757	3.80E-07	14.223616	41	57.595	43.371
chr7	61059828	61063101	0.00018	10.683604	94	57.515	47.754
chr7	61063524	61065985	6.80E-12	12.285167	79	56.57	44.285
chr7	62295393	62300061	9.30E-10	9.835525	145	57.953	47.551
chr7	62300083	62302369	5.90E-12	13.481906	55	55.708	42.226
chr7	62309791	62314587	6.00E-30	12.63325	152	58.652	46.018
chr7	62354666	62357272	4.50E-18	13.994363	92	63.105	49.11
chr7	62402321	62403199	2.10E-07	15.043467	30	56.539	41.495
chr7	62441351	62447606	1.10E-25	11.56426	162	57.376	45.812
chr7	62448063	62455039	1.30E-12	13.776506	207	56.813	44.56
chr7	68126332	68126780	0.0024	15.006625	12	50.18	35.174
chr7	75779600	75779790	0.0013	14.347973	11	47.415	33.067
chr7	86171656	86173046	0.0011	12.767405	21	54.641	41.874
chr7	86809501	86810463	0.0033	14.581085	23	47.756	33.175
chr7	88834245	88835033	0.038	16.374042	17	51.173	34.799
chr7	88843475	88843887	0.012	16.134167	10	62.142	46.008
chr7	89262887	89263887	0.021	15.941123	15	50.888	34.947
chr7	89306226	89306805	0.012	15.17732	11	46.465	31.288
chr7	89428583	89429507	0.045	12.307693	14	37.999	25.692
chr7	89515619	89517032	0.0002	17.701249	21	57.244	39.542
chr7	89975690	89976769	0.024	18.297461	15	48.462	30.164
chr7	90030122	90031641	3.90E-05	17.876657	18	48.152	30.276
chr7	104640200	104641871	0.0069	12.929602	21	44.851	31.922
chr7	104906089	104906308	0.04	21.436565	10	61.491	40.054

chr7	108912673	108913757	0.0032	16.473384	14	54.347	37.874
chr7	109267586	109268003	0.00028	15.028909	21	70.021	54.992
chr7	110401281	110402293	0.025	13.315378	32	62.097	48.782
chr8	3590053	3592470	0.0045	12.297073	32	62.755	50.458
chr8	4438861	4439489	0.0056	14.722599	20	73.09	58.367
chr8	4634379	4635878	0.0035	14.967162	24	74.414	59.446
chr8	4698951	4700299	3.70E-05	17.472155	16	69.204	51.732
chr8	4740587	4741640	0.013	18.765845	11	65.779	47.013
chr8	4802771	4804119	6.20E-06	16.689276	22	56.629	39.94
chr8	4846834	4848012	0.028	12.361728	20	75.924	63.563
chr8	4897253	4898181	0.018	14.631465	18	77.798	63.166
chr8	5094248	5095761	0.049	13.670239	18	78.495	64.825
chr8	5253683	5255628	0.015	11.067392	36	72.596	61.529
chr8	5538341	5539876	0.014	16.368557	19	66.499	50.13
chr8	5597651	5598449	0.0011	18.271537	13	75.506	57.234
chr8	63961206	63962650	1.20E-05	16.166375	28	67.804	51.638
chr8	63971022	63974975	2.70E-09	14.223729	63	60.721	46.497
chr8	64234324	64235432	0.015	13.292842	24	62.012	48.719
chr8	93672352	93674901	0.0015	9.275684	67	57.631	48.356
chr8	117109466	117110828	0.0022	13.777677	22	53.214	39.436
chr8	131126178	131128234	0.038	11.882501	28	60.306	48.424
chr8	134145544	134146383	0.016	17.426747	12	49.314	31.887
chr8	135542169	135542792	0.00051	18.244063	15	64.93	46.686
chr8	135547925	135548783	0.0016	13.635162	22	67.803	54.168
chr8	135552250	135553433	0.021	18.439406	14	73.459	55.019
chr8	135561697	135561920	0.025	20.76994	10	68.428	47.658
chr8	135563261	135565186	8.00E-08	15.773349	35	69.476	53.702
chr8	135588671	135590515	3.00E-07	17.469815	28	68.211	50.741
chr8	135601811	135602771	0.045	14.79996	17	59.096	44.296
chr8	135616410	135617786	0.0012	18.402734	18	72.277	53.874
chr8	135622945	135624123	0.011	15.796435	19	70.567	54.77
chr8	135649615	135650287	0.00087	16.879332	13	82.339	65.459
chr8	135654941	135656414	0.00034	15.761221	19	65.329	49.567
chr8	136048511	136050130	3.00E-06	16.362551	22	59.823	43.461
chr8	136057223	136058175	3.00E-05	23.599522	12	67.854	44.255
chr8	136081312	136081669	0.00044	21.238056	12	55.704	34.466
chr8	136143096	136144853	3.00E-07	16.76387	23	59.285	42.521
chr8	136244334	136246168	0.00013	15.892843	26	63.568	47.675
chr8	136272732	136273310	0.042	16.966484	13	56.734	39.768
chr8	136275456	136277471	5.60E-06	18.799972	20	57.965	39.165
chr8	136300448	136302961	3.80E-11	20.518715	25	64.571	44.052
chr8	136304842	136305587	0.00027	21.188054	12	71.278	50.09
chr8	136307539	136308962	4.30E-10	17.502366	29	58.319	40.816
chr8	136314901	136317319	0.0013	13.294568	29	63.179	49.885
chr8	136326802	136328804	3.40E-06	16.949095	25	59.314	42.365
chr8	136383965	136385040	0.0097	21.604146	12	59.62	38.016
chr8	136393900	136394812	0.001	19.139012	16	57.615	38.476
chr8	136405345	136406639	0.02	15.063897	15	49.517	34.454
chr8	136411090	136412057	0.042	17.371881	12	55.573	38.201
chr8	136414526	136415592	3.10E-05	20.109258	16	58.423	38.313
chr8	136471378	136472748	4.00E-05	17.400388	22	63.262	45.862
chr8	136473458	136474572	1.10E-05	20.709791	15	66.392	45.682
chr8	136621133	136621711	0.04	18.750454	10	54.62	35.87
chr8	136667066	136668088	1.10E-08	20.895921	19	60.286	39.39
chr8	136673983	136675282	0.00069	16.338087	23	59.855	43.517
chr8	136693036	136693740	0.0017	22.847647	10	71.809	48.961
chr8	136863850	136865632	7.10E-07	18.577389	26	53.749	35.171
chr8	137032191	137033596	0.0017	15.262402	23	55.44	40.178

chr8	137092222	137094071	0.00052	16.747697	23	49.404	32.657
chr8	137381123	137382538	0.01	16.090775	22	46.531	30.44
chr8	137546048	137546473	0.023	16.879439	14	59.749	42.87
chr8	137565703	137566863	0.0013	15.781798	17	50.421	34.639
chr8	137579473	137581840	0.0036	15.879865	23	57.352	41.472
chr8	137791965	137794696	0.049	15.139765	21	54.155	39.016
chr8	137826005	137828570	5.30E-05	13.960675	32	56.458	42.498
chr8	139617115	139618380	0.04	13.294218	26	54.703	41.408
chr9	8438547	8439727	0.0053	17.862557	17	77.809	59.947
chr9	8473543	8474694	0.0014	18.92907	15	70.267	51.338
chr9	8490743	8491382	0.00074	20.319133	12	68.563	48.244
chr9	8527886	8532421	2.60E-09	14.919012	44	66.457	51.538
chr9	8586735	8588134	7.10E-05	17.219913	18	69.735	52.515
chr9	25287504	25289789	0.015	10.987941	35	58.213	47.225
chr9	40925348	40926095	0.0093	15.466745	18	60.756	45.289
chr9	99670013	99672213	0.037	11.121898	39	65.974	54.852
chr9	100812135	100813280	0.006	20.375054	12	51.945	31.57
chr9	100832638	100833532	0.038	12.93572	19	75.043	62.108
chr9	118019815	118021002	0.0074	17.153216	17	65.734	48.581
chr10	1980533	1982205	0.0048	14.381093	23	63.983	49.602
chr10	2600265	2601663	0.0032	14.54486	21	61.212	46.667
chr10	2648882	2650106	0.047	13.414368	22	64.579	51.165
chr10	6633631	6634762	0.027	16.38863	14	54.772	38.383
chr10	9037954	9038320	0.0014	18.184308	10	61.917	43.732
chr10	9921593	9923287	5.70E-06	17.109857	24	61.186	44.076
chr10	9929546	9930551	0.012	18.45255	14	59.635	41.182
chr10	10018834	10019693	0.03	18.527396	10	61.553	43.025
chr10	10046564	10047779	0.0041	20.901221	12	62.624	41.723
chr10	10323785	10324637	0.011	19.696073	13	75.258	55.562
chr10	10707243	10708151	0.03	21.498535	10	60.225	38.727
chr10	10986451	10989553	5.20E-06	14.096191	35	64.743	50.646
chr10	11069753	11071192	0.00034	15.283779	24	64.954	49.67
chr10	11084251	11085421	3.80E-05	18.729906	21	72.588	53.858
chr10	11099038	11099696	0.0014	18.70146	14	61.875	43.174
chr10	11112188	11112834	0.0051	20.849718	12	70.854	50.004
chr10	11114523	11116170	0.019	15.343684	18	64.916	49.572
chr10	20096924	20098089	0.024	12.994439	26	78.282	65.288
chr10	38484749	38486572	7.90E-09	14.929487	37	53.526	39.099
chr10	38486945	38490766	3.40E-31	13.766053	78	51.987	38.221
chr10	38490781	38498638	4.10E-18	10.185851	176	55.401	45.464

chr10	38502689	38505020	3.40E-15	14.625992	52	53.214	38.588
chr10	38505039	38509783	4.50E-15	10.641794	107	53.28	42.891
chr10	38522527	38527229	4.60E-21	11.087976	101	51.912	40.824
chr10	38576534	38582936	8.00E-37	11.982174	136	53.769	41.787
chr10	38587497	38590751	0.0004	12.580369	97	61.813	51.498
chr10	38787679	38796838	2.30E-05	11.822665	194	57.7	46.6
chr10	38802683	38805079	0.0012	13.49763	61	66.319	54.568
chr10	38805583	38810870	4.50E-20	11.323779	113	53.243	41.92
chr10	41869489	41871832	0.0023	10.352986	27	42.024	31.672
chr10	41872326	41877143	8.40E-15	11.286767	61	46.782	35.495
chr10	41885715	41888534	2.20E-10	12.402507	33	44.649	32.246
chr10	41894126	41896439	2.70E-05	11.149787	26	44.38	33.23
chr10	41899162	41904274	5.00E-21	11.843613	56	40.811	28.968
chr10	41905013	41906871	5.20E-07	12.33026	27	42.995	30.665
chr10	42088363	42089732	2.00E-05	10.638758	47	63.68	53.041
chr10	42298809	42300042	1.20E-07	12.160503	33	61.334	49.174
chr10	42300436	42308519	2.60E-36	11.546971	159	59.305	47.758
chr10	42309242	42320764	1.10E-28	11.458448	224	60.077	47.896
chr10	42320838	42321583	0.023	16.911933	10	57.06	40.148
chr10	51266263	51268229	0.031	10.748105	30	63.735	52.987
chr10	56981282	56981704	1.50E-05	20.249259	12	64.122	43.873
chr10	57368761	57369451	0.0052	16.639541	18	59.442	42.803
chr10	57467557	57468788	0.042	14.884865	19	55.637	40.752
chr10	60690112	60690680	0.013	21.0429	10	72.24	51.197
chr10	64696625	64697306	0.0003	18.212065	15	52.081	33.869
chr10	64701817	64703754	0.012	14.040017	20	56.839	42.799
chr10	81776525	81777733	0.021	15.967445	15	50.673	34.705
chr10	81829550	81830321	0.019	20.654015	12	60.99	40.336
chr10	82565793	82566817	0.018	13.435575	20	54.157	40.721
chr10	107203599	107204408	0.016	16.660328	12	60.123	43.462
chr10	107246395	107246657	0.013	20.665423	10	66.588	45.922

chr10	107392135	107394077	4.20E-05	15.672186	23	58.517	42.845
chr10	107395909	107397236	0.041	13.829455	27	60.661	46.832
chr10	108047413	108047954	0.003	16.45439	13	70.471	54.016
chr10	108993136	108993934	0.0013	17.454597	15	60.082	42.627
chr10	109284617	109286322	0.016	14.694698	20	54.523	39.828
chr10	130918234	130919586	0.0036	11.382194	21	48.655	37.273
chr11	4656863	4657565	0.0012	23.057734	10	66.55	43.492
chr11	5501336	5502906	0.048	14.146264	17	56.882	42.736
chr11	21230491	21231746	0.0031	16.888421	17	56.91	40.021
chr11	21305931	21307474	0.005	14.774167	18	51.573	36.799
chr11	29760061	29760781	0.045	12.540436	13	43.554	31.013
chr11	40493495	40494467	0.041	18.085101	11	54.703	36.618
chr11	56524209	56525455	0.0096	14.302616	18	55.757	41.454
chr11	56612476	56614201	0.018	13.01916	22	53.959	40.94
chr12	16892461	16894305	0.0088	13.694733	33	58.704	45.01
chr12	67191618	67192491	0.0098	15.518221	14	54.592	39.074
chr12	72441714	72442264	0.037	19.293849	12	62.403	43.109
chr12	87911242	87912169	1.20E-05	15.569939	22	61.832	46.262
chr12	93168294	93168469	0.0084	14.951122	10	29.276	14.325
chr12	102556551	102557050	0.0074	17.635881	10	54.451	36.815
chr13	77937082	77938987	0.0031	12.966231	23	62.595	49.629
chr13	83001019	83002810	0.018	12.298587	30	66.059	53.76
chr13	108287507	108288955	0.0021	13.438485	20	59.403	45.964
chr13	109931009	109931511	0.004	18.482924	13	68.38	49.897
chr14	39817122	39817484	0.0039	15.025545	13	49.608	34.582
chr14	40266840	40267686	0.042	22.760153	10	71.355	48.594
chr14	40273595	40274713	0.021	19.03606	10	53.179	34.143
chr14	41261056	41261776	0.013	16.002227	16	55.186	39.184
chr14	43923478	43924240	0.00052	16.574186	14	48.024	31.45
chr14	44267634	44268731	0.011	15.785994	19	62.689	46.903
chr14	47379280	47380630	0.025	15.512081	14	53.744	38.232

chr14	47391134	47392334	0.031	14.220981	16	52.432	38.211
chr14	47504029	47504783	0.00023	15.313649	15	51.612	36.298
chr14	47522743	47524867	9.90E-08	13.266202	33	48.895	35.629
chr14	47591529	47594147	0.0094	9.488291	32	49.798	40.31
chr14	48422032	48422582	0.00018	14.639601	16	47.564	32.924
chr14	48790247	48791270	0.0063	13.014991	16	42.083	29.068
chr14	48875024	48875873	1.00E-06	19.935782	16	53.545	33.609
chr14	48978438	48978832	0.00035	22.718273	10	70.326	47.607
chr14	48985757	48987278	0.043	13.744513	17	51.302	37.558
chr14	49011148	49012826	0.00031	13.683308	20	43.324	29.64
chr14	49031069	49032759	0.047	13.571037	18	52.613	39.042
chr14	49042280	49043455	0.023	15.984253	13	51.715	35.731
chr14	49068469	49069136	0.00046	18.355826	12	56.651	38.295
chr14	49143201	49144576	0.029	16.475249	15	54.202	37.727
chr14	53181663	53181918	0.035	15.615201	10	52.796	37.181
chr14	79216450	79217432	0.0088	19.247494	11	56.996	37.749
chr14	79403852	79404898	0.0019	16.753715	16	52.945	36.192
chr14	80016287	80016968	6.20E-05	19.889796	13	65.041	45.152
chr14	81981427	81983306	7.30E-05	16.070965	25	63.456	47.385
chr14	82098243	82099045	4.60E-07	21.471775	12	63.852	42.38
chr14	82433686	82434753	0.0032	16.279829	19	61.273	44.993
chr14	82987460	82988796	0.00063	17.701637	16	52.522	34.82
chr14	83026313	83026910	0.022	14.078406	18	74.484	60.406
chr14	83139896	83140195	0.031	15.23961	10	73.727	58.487
chr14	83238465	83239629	0.0016	14.132261	19	69.118	54.985
chr14	96731095	96731996	0.00094	17.062062	17	57.479	40.417
chr14	96950623	96952777	0.012	12.956471	32	73.291	60.335
chr14	98623561	98624270	3.60E-08	19.598186	15	59.27	39.671
chr14	99744536	99744774	0.00051	13.471533	13	30.846	17.375
chr14	101309635	101311403	4.10E-05	12.659016	29	56.425	43.766
chr15	23564797	23565570	5.20E-05	12.215919	23	51.346	39.13

chr15	54716003	54717183	0.035	13.833367	23	51.103	37.27
chr15	97370090	97370677	0.027	17.237117	17	57.071	39.834
chr16	34084650	34086455	0.00079	12.936214	34	52.035	39.098
chr16	34627375	34628845	0.00055	10.756957	50	54.688	43.931
chr16	34660991	34663987	0.0015	13.273699	98	57.337	45.914
chr16	34664397	34666356	0.0016	11.313763	75	59.937	50.698
chr16	34666416	34668819	2.20E-08	10.467008	91	55.583	45.116
chr16	34696451	34701259	5.30E-05	9.499628	154	58.083	48.916
chr16	34722806	34725070	4.40E-05	10.435987	75	59.809	49.373
chr16	34735528	34737226	7.90E-09	14.665671	48	57.954	43.288
chr16	34737310	34744840	0.006	9.638326	257	59.576	50.382
chr16	34751064	34753381	0.0063	12.493669	82	61.855	50.206
chr16	34762148	34763469	0.0026	11.396003	44	57.507	46.111
chr16	34766080	34769056	0.015	10.862776	107	64.467	54.515
chr16	34903453	34904560	0.0046	13.044494	41	59.205	46.16
chr16	36130341	36133454	5.00E-06	10.704164	98	59.244	48.212
chr16	36140267	36143136	2.60E-29	13.487119	101	58.802	45.314
chr16	36167431	36170414	1.70E-13	11.858108	93	56.973	45.115
chr16	36176472	36181285	0.0012	11.601921	155	58.499	47.883
chr16	36184124	36201829	0.032	12.622551	639	61.54	52.248
chr16	36232235	36234480	8.30E-08	9.832712	78	58.012	48.179
chr16	36243650	36246255	7.10E-06	11.681445	79	61.487	51.35
chr16	46404347	46407119	0.0024	12.977902	103	58.578	46.145
chr16	46410531	46414582	1.30E-11	11.538905	121	57.47	45.061
chr16	46417255	46419324	0.0037	10.619746	83	58.93	47.524
chr16	46419359	46422440	1.60E-30	15.675434	106	59.906	44.231
chr16	46464359	46465076	5.90E-05	13.967524	25	55.839	41.872
chr16	46465091	46466592	0.0072	10.517183	59	58.44	46.636
chr16	72493250	72494459	0.034	12.318972	20	72.293	59.974
chr17	21864715	21870182	9.40E-15	9.442746	139	61.416	51.973
chr17	21987250	21989847	3.70E-07	11.97998	33	42.923	30.943

chr17	26794521	26798140	1.20E-05	8.53058	103	63.625	55.095
chr17	26823259	26828164	0.00024	8.028997	104	59.103	51.074
chr17	26855510	26859621	1.20E-07	9.484419	96	58.893	49.409
chr18	13672797	13674227	0.0096	13.815242	19	68.313	54.498
chr18	14790999	14793579	0.0043	11.887166	26	58.698	46.811
chr18	14794614	14794739	0.0049	19.32853	12	69.646	50.318
chr19	30224397	30224799	0.00067	-14.632211	23	32.386	47.018
chr19	56788299	56789649	0.02	12.206252	17	54.379	42.172
chr20	10670512	10670935	0.023	12.409415	12	27.3	14.891
chr20	30812565	30813196	0.0034	13.499701	28	63.156	49.657
chr20	31081766	31082887	1.70E-05	13.34387	30	51.245	37.901
chr20	31094112	31096736	0.047	7.264823	51	41.938	34.674
chr20	31157151	31158907	0.043	8.543982	29	38.7	30.156
chr20	31161687	31169700	9.00E-08	10.563829	179	49.869	39.814
chr20	31169730	31173374	1.40E-12	10.314257	71	46.417	36.103
chr20	31181460	31182999	6.30E-09	16.03848	24	48.245	32.207
chr20	31187609	31190441	1.30E-05	10.697578	63	46.824	35.258
chr20	31190879	31194504	2.60E-12	11.241877	83	52.614	41.372
chr20	31228579	31234272	4.10E-20	11.079758	121	50.62	39.541
chr20	42280998	42283231	0.035	11.767341	27	53.068	41.301
chr20	42528672	42529346	0.022	13.482885	21	60.421	46.938
chr20	44670784	44671132	0.0011	21.123733	10	58.915	37.792
chr20	52602956	52603390	0.03	16.431162	10	61.942	45.511
chr20	55774730	55775565	0.025	15.141648	17	54.074	38.933
chr20	60163665	60164802	3.20E-06	16.86452	20	53.549	36.685
chr20	60277705	60278897	0.043	15.001988	21	63.076	48.074
chr21	7916532	7918586	0.0021	8.979242	41	53.631	44.652
chr21	7946421	7950240	0.0043	8.742186	50	44.521	35.779
chr21	10653303	10658275	7.20E-08	9.354614	90	51.515	42.092
chr21	10658329	10664492	6.00E-22	11.188148	100	53.148	41.96
chr21	10670466	10675992	0.00066	12.933649	104	50.559	40.044

chr21	10676655	10680655	6.30E-17	10.895576	67	48.935	38.039
chr21	10680683	10694896	0.0065	8.328619	256	54.392	45.551
chr21	10708506	10713196	0.00072	8.220418	68	49.787	41.566
chr21	10735137	10738675	0.0035	8.896205	48	48.115	39.219
chr21	13516374	13518174	0.0017	12.375811	27	65.245	52.869
chr21	19770301	19772098	0.0035	12.049468	22	53.667	41.617
chr22	10716932	10719608	0.0021	7.605165	53	48.562	40.957
chr22	10728556	10729832	3.90E-05	11.246976	27	53.274	42.027
chr22	16267349	16268301	1.60E-05	12.782853	33	55.895	43.113
